# The biomechanics of wounds at physiologically relevant levels: Understanding skin's stress-shielding effect for the quantitative assessment of healing

**DOI:** 10.1016/j.mtbio.2024.100963

**Published:** 2024-01-17

**Authors:** Sara Medina-Lombardero, Connor Bain, Laura Charlton, Antonella Pellicoro, Holly Rocliffe, Jenna Cash, Robert Reuben, Michael L. Crichton

**Affiliations:** a School of Engineering and Physical Sciences, Heriot-Watt University, Edinburgh, EH14 4AS, United Kingdom; b School of Engineering, University of Edinburgh, Edinburgh, EH9 3RF, United Kingdom; c Centre for Inflammation Research, University of Edinburgh, Edinburgh, EH16 4TJ, United Kingdom

**Keywords:** Biomechanics, Skin, Wound healing, Digital image correlation, Tensile testing, Viscoelasticity, Collagen alignment

## Abstract

Wounds are responsible for the decrease in quality of life of billions of people around the world. Their assessment relies on subjective parameters which often delays optimal treatments and results in increased healthcare costs. In this work, we sought to understand and quantify how wounds at different healing stages (days 1, 3, 7 and 14 post wounding) change the mechanical properties of the tissues that contain them, and how these could be measured at clinically relevant strain levels, as a step towards quantitative wound tracking technologies. To achieve this, we used digital image correlation and mechanical testing on a mouse model of wound healing to map the global and local tissue strains. We found no significant differences in the elastic and viscoelastic properties of wounded vs unwounded skin when samples were measured in bulk, presumably as these were masked by the protective mechanisms of skin, which redistributes the applied loads to mitigate high stresses and reduce tissue damage. By measuring local strain values and observing the distinct patterns they formed, it was possible to establish a connection between the healing phase of the tissue (determined by the time post-injury and the observed histological features) and the overall mechanical behaviour. Importantly, these parameters were measured from the surface of the tissue, using physiologically relevant strains without increasing the tissue's damage. Adaptations of these approaches for clinical use have the potential to aid in the identification of skin healing problems, such as excessive inflammation or lack of mechanical progression over time. An increase, decrease, or lack of change in the elasticity and viscoelasticity parameters, can be indicative of wound state, thus ultimately leading to improved diagnostic outcomes.

## Graphical abstract

**Figure:**
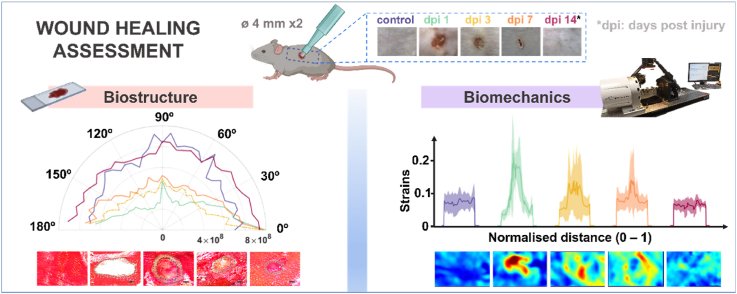

## 1 Introduction

Chronic (non-healing) wounds (CW) are a global socio-economic burden causing quality-of-life issues such as pain, emotional distress, and, in more severe cases, can lead to life-threatening situations such as amputation or sepsis. In 2020 it was estimated that the UK's NHS spent around £8.3 billion per year to tackle them [1], a number that has seen a steadily rise every year [2]. Yet, CW are still perceived as a symptom of other conditions (i.e., comorbidities) rather than as a disease in themselves [3], and are, consequently, an “underreported health issue” [4]. Due to the lack of attention this problematic receives, CWs are frequently classed as a hidden or silent epidemic [5].

There is a clear need to improve wound assessment strategies, and to this end, there has been a rise in the development of point-of-care devices and wearable sensors in the recent years, to offer non-invasive and quantitative ways to monitor skin's health [[6], [7], [8], [9], [10], [11], [12]]. Whilst most of these commonly focus on biological or physical parameters (glucose, pH, moisture content, etc.), measuring the mechanical properties of skin offers a powerful way of evaluating dermatological conditions, as this not only evaluates healing on the surface of the tissue, but also by taking into account sub-surface changes. Different mechanical techniques allow measurements at different tissue scales (e.g., bulk or local measurements, at a tissue or cellular scale, etc.), which is of particular interest for evaluating composite tissues with the complexity that skin has. Emerging mechanical systems to monitor changes to skin include acoustic sensors, imaging for area analysis or elastography (compressional or shear wave) [8,10,[13], [14], [15], [16]]. Yet, for clinically implementing such technologies, it is often recognised that the underlying understanding of wound healing mechanics is still relatively limited [17].

In wound healing, there are generally 4 physiological stages (haemostasis, inflammation, proliferation and remodelling) where clotting, material production, removal and exchange (respectively) will cause progressive mechanical strengthening [18]. Specific actions during this time include the arrival of platelets for clot formation, innate immune cells that fight pathogens, and the deposition and remodelling of collagen fibres that reconstruct the extracellular matrix (ECM) [19]. Whilst these bring transient complexity when measuring a single change in a wound, the progressive changes provide a new opportunity to track the local progress within a wound non-invasively with mechanical methods. However, this is a challenge, when biomechanical assessments of healthy skin alone yield widely varying mechanical results [20].

To date, the mechanical properties of wounds have been investigated widely, but to the authors' knowledge the way in which stress is balanced in physiologically relevant strains is still under-researched. In general, previous works attribute more importance to wounded skin failure properties, using forces which are generally not experienced in a live individual's skin. They commonly measured the strength and extensibility of tissues at a macroscale (e.g. Refs. [[21], [22], [23]]), by stretching excised skin containing a wound, but often without including the adjacent tissue margins (which have a well-known role in the re-epithelisation of wounds and, hence, in their healing [24,25]). These studies found correlations between the degree of healing (mainly represented by the amount, thickness and orientation of collagen in the wounds), the peak force needed to break the specimens, and the extension reached before the fracture. Expectedly, skin has been found to have a lower strength and extensibility at early healing phases, which gradually increase with time as the tissue recovers [26,27]. It has also been noted that fully healed tissues would only recover 80 % of their original strength, due to persisting microstructural changes in the long term [18]. Despite these important findings, these works were destructive to the tissue, and therefore removed from the physiological range needed for clinical diagnostics/treatment.

In recent years, there has been a shift in the paradigm of soft tissue testing, advancing towards the use of more physiologically relevant strategies. To achieve this, the newer methodologies implement traditional bulge and suction tests, indentation, compression and tensile tests, etc., but now utilising a lower range of forces and strains [[28], [29], [30], [31], [32]]. A common issue encountered by such mechanobiological studies is the fact that skin is highly non-linear, anisotropic, and is subjected to many sources of variability (both intrinsic, due to the nature of the tissue, but also extrinsic, due to the testing methods themselves and environmental factors) [33]. Thus, many of these authors complement these measurements with non-contact imaging systems and finite element models, to be able to further characterise the tissues locally and validate their findings.

Although the application of these methods is growing in the soft tissue field, only a handful of studies have employed these advances (experimentally) to the study of healing wounds (i.e., as opposed to scars). Good examples of these are the studies of: Chao et al. [34], which provided local stiffness values measured with air jet indentation in the periphery and core of wounds (on days 0, 3, 7, 10, 14, and 21 post injury, in rat skin); Pensalfini et al. [35], which presented a wider field characterisation of ex-vivo tissue using full-field optical methods coupled with tensile testing (on days 7 and 14 post injury, in mice skin); and Wietecha et al. [36], which adapted the techniques from Pensalfini et al. and used them on live mice (on days 3, 5, 7, 10, 14 and 21 post injury). A general agreement from these works is that wounds present a higher stiffness in their core when compared to a baseline (i.e., “healthy counterpart”) value. However, whilst the first two studies only partially characterised the tissue (due to using a discrete point-measurement approach in the first case, and to the partial exclusion of wound adjacent areas of the specimens in the second), the latter provided a visual representation that averaged strains in a way that masked local stress adaptations around the wound perimeter. The visualisation and characterisation of the whole tissue heterogeneity is relevant to better understand the shielding mechanisms that wounds “activate” when subjected to wider stressors. Furthermore, there still remains the need to link the surface and sub-surface mechanical properties in wound healing in a way that could provide benefit for wearable technology or diagnostics.

In this work, we sought to further assess both the stress distributed by skin during wound healing, and its corresponding microstructural adaptations over time. We employ digital image correlation and mechanical testing on a mouse model of wound healing in order to map the global and local tissue strains, to quantify the influence of wounds in their surrounding tissues. Recognizing the lack of viscoelasticity considerations by other studies in early healing wounds (with only Hamilton et al. [27] found as a reference for day 10 wounds), we also included analysis of the bulk relaxation properties of wounds, as the moisture content of each is likely to change throughout the healing, potentially becoming an additional mechanical biomarker. Finally, by analysing histological data, we are able to correlate each wound stage to a unique alignment coefficient, which in turn can be associated to the different mechanical disturbance patterns measured locally. Doing this provides insights into how strain evaluations can support decisions such as optimal sensor placement in the design phase, or to inform clinical surgical repair (i.e. suturing).

## 2 Materials and methods

### 2.1 Animals

All experiments were conducted with approval from the University of Edinburgh Local Ethical Review Committee and in accordance with the UK Home Office regulations (Guidance on the Operation of Animals, Scientific Procedures Act, 1986) under PPL PD3147DAB. Experiments at Heriot-Watt University were conducted under ethics number 18/EA/MC/1.

C57 B l/6JCrl mice (Charles River, Tranent, UK) were maintained in conventional cages on a 12:12 light:dark cycle with *ad libitum* access to standard chow and water under a SPF environment. Animals were housed 3–5 per cage in a temperature (22–24 °C) and humidity controlled room. Environmental enrichment was provided in the form of dome homes, a tunnel and chew sticks. Health checks were performed on all animals prior to and at each wounding time point, including baseline weight measurements. Only animals that were not involved in previous procedures or had no further disruptions or defects in the wounded area were used for experiments.

### 2.2 Murine dorsal skin wounds

Mice (7–9 weeks old) were randomly assigned a wounding group and anaesthetized with isoflurane (Zoetis, Leatherhead, UK) by inhalation. Buprenorphine analgesia (0.05 mg/kg, s. c, Vetergesic, Amsterdam) was provided immediately prior to wounding and dorsal hair was removed using a Wahl trimmer. Two full-thickness excisional wounds were made to the shaved dorsal skin using sterile, single use 4 mm punch biopsy tools (Selles Medical, Hull, UK). Wounds were photographed with a Sony DSC-WX350 and a ruler immediately after wounding and at cull. Mice were housed with their previous cage mates in a 28 °C warm box (Scanbur, Denmark) overnight following wounding, with paper towels used as bedding to avoid sawdust entering the open wounds. Dome home entrances were enlarged to prevent animals scraping their dorsal skin wounds. Animals were moved into clean conventional cages at 22–24 °C the following morning. Animals were culled at 1, 3, 7 and 14 days post-wounding by rising concentrations of CO_2_ by inhalation and cervical dislocation.

### 2.3 Skin harvesting and preparation for testing

Dorsal hair was removed again at time of harvest using a Wahl trimmer. Noting that the skin has a tendency to shrink after removal, we sought to minimise sample variability by using an adhesive stencil to mark the *in vivo* dimensions before excision. As opposed to humans and pigs, mouse skin is loose in nature and its tension can be modified inadvertently even before excision. Thus, care was taken to avoid over-stretching the skin tissue during the cervical dislocation.

Sample dimensions were of 20 mm gauge length, 10 mm width, and 40 mm total length. These were transferred to skin with a Sharpie pen (Sharpie, USA). A total of 10 samples per time point were obtained.

The skin was removed with scissors and forceps and placed on a paper moistened with phosphate buffer saline (PBS) for transportation (not submerged). Samples were stored at 4 °C and brought to room temperature by removing from the fridge for 1hr before testing (∼22 °C). All tests were carried out within 48 h post-mortem.

Hair removal cream (Veet, UK) was used to remove excess hair. The samples were placed flat on a cutting mat, held at the original dimensions and then cut with the aid of a cutting die, to minimise shear stresses and the overstretching of the wounds during skin manipulation.

The top (epidermal) surface of the samples was lightly dried with compressed air at low pressure and a random speckle pattern was applied on top by flicking the bristles of a toothbrush coated in an alcohol-based ink (Fluids Alcohol Ink Midnight, Octopus Office, Germany).

The samples were then placed on dry paper to keep them from twisting or changing dimensions while positioning them within the testing equipment. Once clamped, a few drops of PBS were applied on the paper, which helped its detachment from skin and removal from the testing rig, as well as slightly remoisturising the sample from underneath (see Supplementary Fig. S1). No PBS was added to the top of the skin sample to avoid disturbing the speckle pattern applied. No further rehydration was provided during the testing; however, sample dryness was not observed at the end of the tests as the moisture initially provided to the dermis and the environmental conditions favoured the experiment.

### 2.4 Tensile testing with digital image correlation

Uniaxial tensile tests were carried out with an ElectroForce 200 N TestBench (TA instruments) in a horizontal configuration, with a 10 N load cell. A custom-made clamp with knurled jaws was used to avoid slippage.

Monotonic tests were performed in displacement control at a rate of 1 mm/s (0.05 s-1). The movement was set to stop when the force measured by the load cell reached 0.5 N, which was used as the starting point for subsequent stress relaxation (viscoelasticity) experiments, in which the sample was held for 500 s at that position. Whilst a plateau force might not be achieved at such relaxation intervals, these measurements were deemed representative for future clinical investigations, where shorter times are beneficial when testing *in vivo*.

It was observed that some of the scabs would break up even at such low force levels, yet a minimal displacement of 2.5 mm was desired to obtaining sufficient data for the calculation of elasticity from all test groups.

A camera (Canon EOS 2000D) equipped with a macro lens (Sigma 105 mm f2.8) was used to record the top view during the specimen loading, for local strain calculations. A second camera (Canon EOS 2000D) with a compact lens (EF-S 18–55 mm IS II, Canon) was added from the lateral view for measurements of thickness.

### 2.5 Histological analysis

Histology was performed to visualise the microstructural organisation of the tissues. Whilst 3D methodologies to study collagen *in vivo* during loading have been developed in recent years [35,37], a static analysis remains a valid approach for the purposes of the present study.

To preserve skin's biostructures post-testing (i.e., to avoid further relaxation or crimpling of the fibres), the samples were snap frozen within 2 min after the tests finished.

**Fig. 1:**
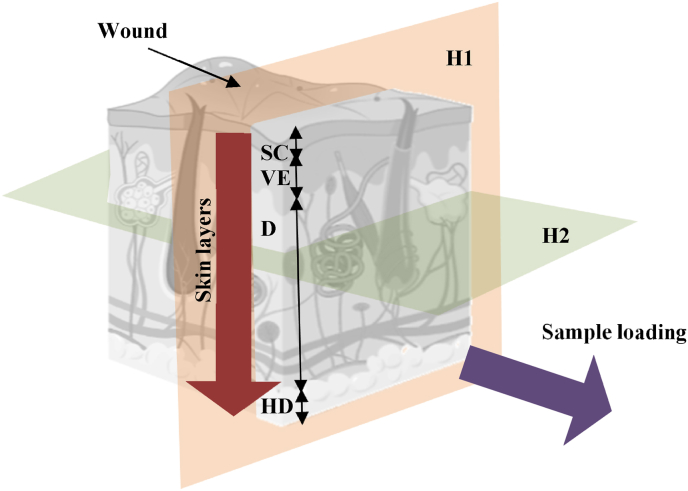
Sectioning planes. H1 corresponds to the lateral view, where all skin layers (Stratum Corneum SC, Viable Epidermis VE, Dermis D, and Hypodermis HD) and other components (i.e., follicles, glands) can be visualised as well as the wound depth and lateral expansion. H2 is the planar approach, which is used to visualise the wounds and tissue structures parallel to the loading plane. Skin schematic from Biorender.

Both a lateral and a planar approach (i.e., in the plane of the skin surface) were taken when sectioning the tissue (see Fig. 1). Thus, microstructural information was obtained both on the same plane as that of the strain maps created with the imaging protocol, and on the plane perpendicular to the wound, where all layers of skin can be observed and the extension of the wounds at each stage can be measured.

To obtain the sections, samples were first cut to a size of approximately 1 cm^2^ around their central area, then placed dermis-side down into the disposable embedding mould (Peel-A-Way Embedding mould, Sigma-Aldrich) which afterwards was filled with a sectioning matrix compound (OCT, Thermo Fisher Scientific). The mould was then placed into a cooled isopentane filled container partially submerged in liquid nitrogen, as described in Ref. [38]. Care was taken to prevent the samples from floating or flipping in the solution, by pinning them down with tweezers while the freezing process was taking place (∼1 min).

The frozen cubes were then sectioned in a cryostat (HM525 Cryostat, Thermo Fisher Scientific), obtaining 10 μm thick skin's slices, which they were fixed with a Formaldehyde solution (the fixation protocol is provided in **Sup**. **Materials**, section 3).

For the visualisation of morphological features (particularly, the collagen mesh in the dermis) the sections were stained with Picrosirius red (PSR), adapting the protocol from Ref. [39] (see **Sup**. **Materials** section 3 for details). To acquire further information of the cellular and extracellular components at the wound site and its vicinity, some of the laterally sectioned samples were stained with Hematoxylin and Eosin (H&E), following the protocol from Ref. [40].

Finally, an optical microscope (Axio Observer, Zeiss) was used to visualise the staining and for automatically stitching the images for the analysis.

### 2.6 Data analysis

#### 2.6.1 Curve fitting for elasticity and viscoelasticity parameter obtention

Displacement (δ), force (F) and time (t) data were recorded with the ElectroForce equipment. Monotonic tensile test data was fitted with the hyperelastic Ogden model of first order, as recommended by Refs. [[41], [42], [43]]:

(1) $$\sigma \left(\lambda \right)=\mu \left(\lambda^{\alpha }-\lambda^{-\alpha /2}\right)$$

where:

σ is the Cauchy stress, which is calculated as the nominal stress multiplied by the stretch ratio ($\sigma =\frac{F}{A}\lambda$). The area of the samples was calculated by multiplying the width by the thickness of the samples.

λ is the stretch ratio, which can be calculated as λ=1+ε, with $\varepsilon =\delta /l_{0}$, and $l_{0}$ being the initial gauge length of the sample

μ and α are the model fitting parameters: μ is said to correspond to the shear modulus (rigidity), whereas α is a strain hardening component (stiffening effect).

Relaxation data for viscoelasticity evaluation was fitted with a two term Prony series:

(2) $$G\left(t\right)=G_{0}-g_{1}\left(1-{\mathrm{e}}^{-t∕\tau_{1}}\right)-g_{2}\left(1-{\mathrm{e}}^{-t∕\tau_{2}}\right)$$

in this case, the model fitting parameters are:

$g_{1\;}$ and $g_{2\;}$, which correspond to the shear material constants (relaxation magnitudes).

$\tau_{1}$ and $\tau_{2}$, which correspond to the time scales of each Prony term (relaxation times).

t is the time vector recorded by the equipment.

$G_{0}$ is the elastic shear modulus which, in this case, it is obtained from the loading data fitted with equation (1).

All samples were batch processed in MATLAB (v.2021a). Fitting functions were generated with the in-built Curve fitting app, using a non-linear Least Squares method with a maximum of 3000 iterations, and decreasing the tolerance criteria TolFun to 10^−12^ and TolX to 10^−8^, as recommended in Ref. [41].

Whilst the chosen models do not account for the inhomogeneities (or anisotropy) within the samples, the aim of this study was not to accurately “predict” the behaviour of skin, but rather to obtain comparative values from each cohort without *a priori* structural information. To ensure numerical stability, fittings with an r-square value lower than 0.99 were removed from the analysis, as corroborated by visual inspection that those fittings were incongruent with the expected shape of the data (e.g., due to data saving errors, a flat line appeared at times instead of the J shaped curve).

#### 2.6.2 Digital image correlation: local strains

The post-processing of the videos was performed with “ncorr”, an open source MATLAB application [44]. No modification or enhancing was performed on the video frames, other than in-built methods included in the “ncorr” program, the details of which can be found in Refs. [44,45], and a manual cropping to remove the samples’ background and reduce processing times. In general, a pixel in one of our images corresponded to 0.020 ± 0.002 mm (slight variations occurred due to movements of the camera from day to day, which were accounted for in a calibration step) (see **Sup**. **Materials** section 2, for details regarding the validation of this method).

A circular subset of 20 pixel radius was used in the reference configuration with no subset spacing. The subset size was empirically determined after checking that it was the smallest size that could be used with minimal loss in correlation. This was verified at the highest strains, where most gaps appear when the correlation search fails.

The norm of the difference vector cut-off was increased from the default 10^−6^ to 10^−4^, thus relaxing the correlation criteria between consecutive images (“ncorr” default options are defined as “pretty strict” by its own authors [46]). The number of iterations was doubled (from 50 to 100) to increase the chances of finding a solution, which slightly increased the processing times. High strain analysis was enabled, as skin is a hyperelastic material and high deformations were expected (i.e., as compared to other materials such as metals). Automatic propagation of seeds and subset truncation were also enabled. Four computer cores were used simultaneously for the correlation calculations in parallel, to decrease the processing time. In the last step, strain radius was set to 15, which is the smoothing factor by default in “ncorr”.

Green-Lagrangian strains were used as these are calculated with respect to the reference configuration and thus, they allow a spatial comparison (i.e., point to point) of different strain levels.

In-plane direct $\left(\varepsilon_{xx},\varepsilon_{yy}\right)$ and shear $\left(\varepsilon_{xy}\right)$ strains were obtained from the DIC codes, and from these, the equivalent plastic strain $\varepsilon_{eq}$ was calculated with the formula:

(3) $$\varepsilon_{eq}=\sqrt{\frac{2}{3}\varepsilon_{dev}:\varepsilon_{dev}}$$

With $\varepsilon_{dev}=\varepsilon -\frac{1}{3}\;tr\left(\varepsilon \right)∙I$ being the deviatoric strain tensor, ε being the Lagrange strain tensor and I being the identity tensor (all second order tensors).

As with the von Mises stress, the equivalent plastic strain provides a scalar value for each position on the skin's surface. Deviatoric strains are linked to shape changes, whereas hydrostatic ones are correlated to volume changes. Hence, as the equivalent plastic strain is most dependent on the former, these values will be indicative of relative shear strain at each point.

#### 2.6.3 Data extraction from strain maps

**Fig. 2:**
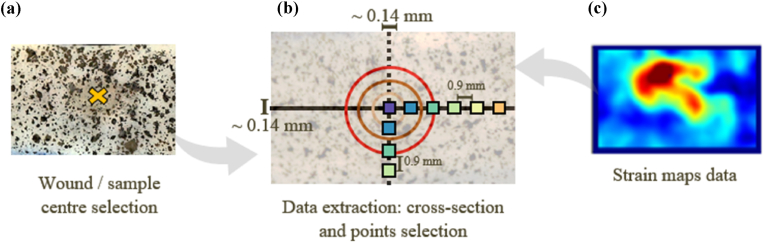
Local data extraction from the strain maps. (a) The centre of the maps is selected from the raw image data rather than the strain maps in (c). (b) Two cross-sections passing through the centre of the strain maps are used to extract quantitative data from each individual sample. The concentric circles in represent the wound size changes at different stages (the larger circle is the initial 4 mm punch). The squares are the discrete points taken across the cross-section lines, each at a distance of 0.9 mm, for statistical comparisons between the regions of each cohort.

To compare the local strains across different wound types, a stripe passing through the centre of the strain maps in both the perpendicular and transversal directions was selected (see Fig. 2 black line for longitudinal, dashed line for transversal measurements). The coordinates of the centre of each sample were chosen manually, using the raw image data instead of the strain maps, to avoid picking points within the higher strain concentration areas on purpose. The stripes were of 7 pixels width (approximately 0.14 mm) and median values were taken at each point along them, to minimise the influence of outliers.

#### 2.6.4 Histological analysis: fibre alignment and density in wounded an unwounded skin

Top views (H2 in Fig. 1) were used to evaluate collagen alignment on the loading plane, and especially to see how the fibres arranged around wounds. The area for the alignment coefficient (from now on, AC) calculation, was selected by following two criteria: first, the aspect ratio of the cropped image had to maintain that of the original image, and second, areas where the boundaries of the sample were visible were removed, to avoid skewing the measurements. The overall directionality of the fibres was studied first, by selecting all the area surrounding the centre of the sample (to obtain a global AC) and second, by selecting two smaller regions of interest (each of 100 × 100 pixels): one adjacent to the visible wound edges, and a second one adjacent to the edge of the sample (far field), to study how the AC locally evolved with the healing. At least 3 samples per each time point were used for the comparisons between days.

To measure fibre alignment a custom-made MATLAB code was used (named FIBRAL). Briefly, image features were first enhanced in the real domain by switching from the “RGB” colour space to the “L*a*b” one, selecting the channels of interest (channel “a”, and positive region of “b” channel) and increasing brightness and contrast. Then, the enhanced images were transformed into the frequency domain using a 2D fast Fourier transformation, and the alignment coefficient was extracted from the intensity values measured at different angles of the corresponding plot (from 0 to 180°). Note that the AC is independent of the direction of the input image, as it just indicates the portion of the fibres that are oriented in one same direction of an image, but does not specify which direction that is. The polar plots are provided for a day-to-day comparison in a chosen image orientation (in this case, cranial to caudal in the horizontal/0° axis), however this is merely for visual purposes. More details about the code can be found in Rocliffe et al. [40] and in the **Sup. Materials (**section 4**)** of this paper.

To quantify the density of collagen fibres in each image, a simple pixel area algorithm was employed. Using the enhanced grayscale image exported through FIBRAL, the total area of fibrous tissue was represented by the fraction of the image with pixel values over 30 – thus excluding any background areas with very minimal red, which in turn indicates the lack of collagen or its very little presence in those regions –, versus the total number of non-zero pixel values on the image (all pixels being originally between 0 and 255).

#### 2.6.5 Statistics for wound staging

The statistical tests used to compare the different wound groups were tailored to each experiment: considering the sample sizes and whether normality in the distributions could be assumed or not, both parametric and non-parametric approaches were taken.

For pairwise comparisons in the alignment coefficients (each group containing 3 samples), a one-way ANOVA test was performed, with a post-hoc Bonferroni correction. Only p-values <0.005 were considered significant, due to the low number of samples used in this analysis and thus, the need for a stronger evidence to reject the null hypothesis.

For comparisons between the global mechanical parameters (μ, ⍺, g1, g2, τ1 and τ2) of each wound and control group (all ≥8 samples), the Shapiro-Wilk test (for n < 50 samples) was first conducted to determine whether the normality of the distributions could be assumed. A one-way ANOVA test was later carried out to find out if the mechanical properties of skin differed in relation to the degree of damage/healing. Finally, Tukey's and Dunnett's tests were performed post-hoc, to evaluate which (if any) differences found were significant (with p < 0.05).

The local analysis of strains consisted in comparing discrete points within one same wound stage, and between points of the same region in different groups (e.g., region 1 in control group vs region 1 in the day 1 wound group). Recognizing the non-normality of the data in regional comparisons, we opted for Kruskal-Wallis test for this last analysis. A post-hoc Dunnett's test was then carried out to evaluate the significance of the findings.

All statistical analysis were carried out in MATLAB (MATLAB v2022a), by using the functions ‘swtest’ for the evaluation of normality, “ttest2” for the pairwise comparisons in the alignment coefficient, “anova1” for multivariance analysis of the mechanical parameters, and “multcompare” for the post-hoc analysis/

## 3 Results

### 3.1 Healing progression: wound morphology and histological assays

**Fig. 3:**
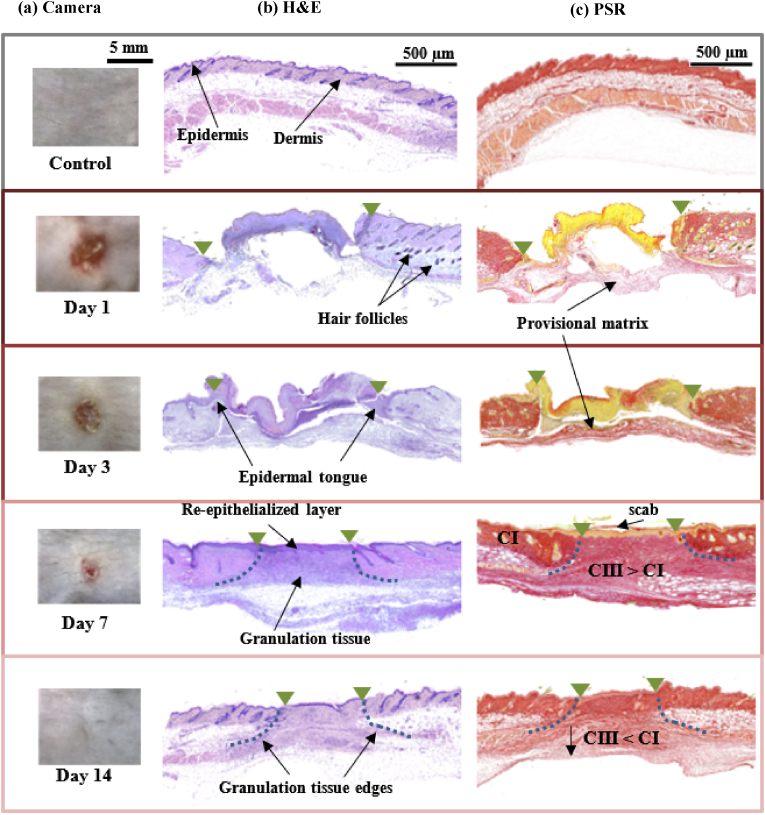
Wound evolution throughout healing. (a) Wound visual appearance before excision from the animal, showing the reduction in size throughout healing. (b) Histological results showing cellular presence within and around the wounds. (c) PSR stained samples, collagen fibres appear in red. Wound margins are marked with green triangles, and granulation tissue edges are marked with a dashed line. CI and CIII refers to collagen types I and III respectively. (For interpretation of the references to colour in this figure legend, the reader is referred to the Web version of this article.)

A representative example of the wounds’ appearance at each time point (pre-excision), is shown in Fig. 3a.

Samples sectioned in the plane perpendicular to loading (i.e., plane H1 in Fig. 1) and stained with PSR are shown in Fig. 3b. Corresponding H&E-stained wound mid-sections are presented in Fig. 3c, for an initial evaluation of the cellular proliferation and other histomorphological features.

One day post-wounding, a provisional matrix is deposited in the wound area (this is seen as a thin layer under a scab). Polymorphonuclear neutrophils (PMNs) start infiltrating the wound, and the area starts to contract, however no reepithelialisation takes place yet. The density of collagen fibres at this stage is calculated as 61.51 % ± 6.97.

On day 3 after the biopsy, all wounds possess a scab and have contracted to approximately 40 % of their original area, as measured by Rocliffe et al. [40] on the same cohort. The so-called epidermal tongue which helps in closing the wounds can be observed on day 3 [47]. The density of collagen fibres now has gone up to 78.03 % ± 10.54.

On day 7, the contraction of the wounds is far more evident (∼17 %), and they start to lose the scab as wound reepithelization completes. Collagen deposition can be observed at this stage (mostly CIII, according to Pensalfini et al. [35]), and the epidermal layer is thicker (60–70 μm) than in healthy tissues (∼20 μm) due to the hyperproliferation of cells in the early repair phases of healing. Collagen density is similar to that on day 3 (76.82 % ± 11.78).

On day 14 the scab is not present anymore, but despite the apparent superficial recovery biological differences remain at deeper levels of skin. This can be observed in the histological analysis (i.e., the granulation tissue can still be appreciated under the surface); macroscopically these changes conform to what is commonly known as a scar. The collagen fibre density at this stage is of 83.99 % ± 5.97; closer to the control density values (99.02 % ± 2.02) but not fully recovered.

### 3.2 Natural collagen arrangement within and around wounds and its response to loading

**Fig. 4:**
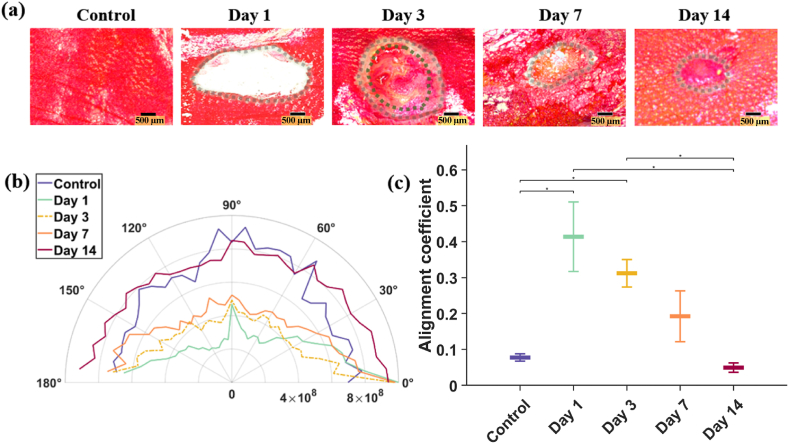
Biostructural arrangement analysis. (a) Picrosirius red staining of representative samples from each day (all scales are 500 μm and wound margins are delineated with a dashed line). Note that resolution in this figure is reduced, refer to sup. materials Fig. S5 for a higher resolution representative example. (b) Corresponding polar plots obtained through FIBRAL, x-axis values are related to the proportion of fibres oriented at each 5-degree bin size. (c) Average alignment coefficients with standard deviation (the closer to 1, the higher the alignment in the wound-adjacent tissue). P-values < 0.005 are shown in the figure. (For interpretation of the references to colour in this figure legend, the reader is referred to the Web version of this article.)

Fig. 4a shows typical results of skin section enface (Fig. 1, plane H2) with PSR staining, to visualise collagen parallel to the loading plane. Fig. 4b shows the polar plots in which the intensity detected at each angle in the frequency domain (proportional to the number of fibres oriented in each direction) is presented.

Whilst the images in the spatial domain might not display evident fibre features to the naked eye, once the images are enhanced and transformed to the frequency domain there appear clear dominant fibre orientations.

In days 1 and 3, based on the polar plots, it can be seen that most fibres in the image (note that lines have been scaled, to ease their visualisation) lean towards the 0–180° line, indicating a high collagen alignment, in this case, in the cranial to caudal direction (day 1 AC: 0.41 ± 0.10; day 3 AC: 0.31 ± 0.04). On day 7, the alignment coefficient (AC 0.19 ± 0.07) has reduced almost by half (46 %) compared to that of day 1.

Finally, on day 14 there is a similar number of fibres at all angles (AC 0.05 ± 0.01), indicating a higher anisotropy in the tissue. This later arrangement is comparable to the one encountered in healthy (control) skin (AC 0.07 ± 0.01), thus suggesting a high degree of microstructural recovery 2 weeks after the wounding.

When looking at the alignment locally (i.e., in wound adjacent vs far field regions), it was found that the alignment coefficients where consistently higher in the regions near the wound than further away from it (these values can be found in **Supplementary materials, section 8**), although they all followed the same trend: more alignment was found in early days in all regions, than later in the healing.

### 3.3 Global elastic and viscoelastic properties of different healing phases

**Fig. 5:**
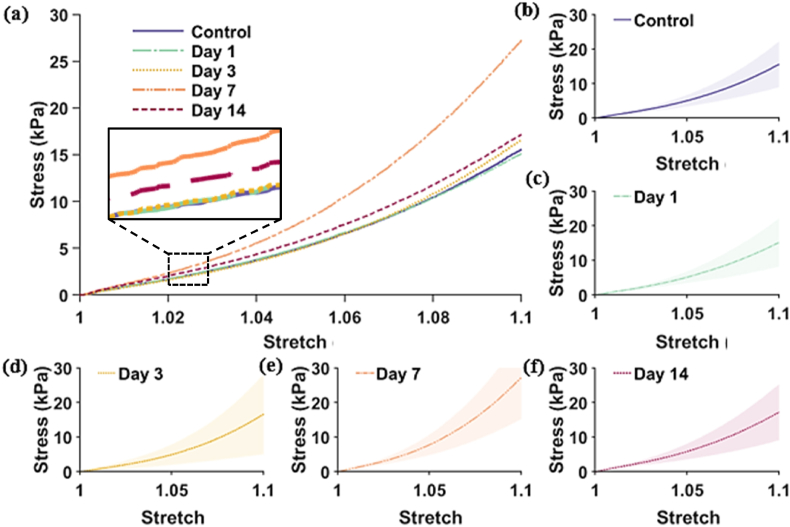
Loading stress-strain curves from tensile tests. The mean loading curves are shown in (a) which are the combined curves from (b)–(f), where individual days 1–7 are presented. ± SD (shaded).

In Fig. 5 the average global stress-strain curves for each day are presented (up until 10 % strain, λ = 1.1). Each curve is also individually plotted showing the standard deviation as a shaded region.

Day 3 had a larger standard deviation (SD: 10.7 kPa), and day 7 presented a steeper curve compared to all the rest. Surprisingly, day 3 and day 1 samples, despite having a larger wound present, presented a similar trend to that of unwounded samples.

**Fig. 6:**
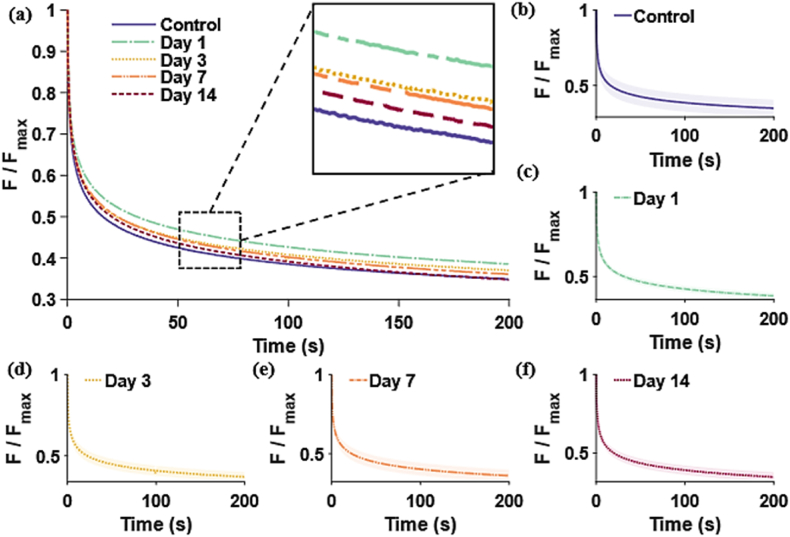
Relaxation curves of wounded skin. Mean curves are shown in (a) which are the combined from (b)–(f) where individual days 1–14 are presented. ± SD (shaded). The relaxation behaviour is similar between all samples.

In Fig. 6 the global relaxation curves for each day are shown. The standard deviation is again displayed as a shaded region in individual curves.

Once more, there is not an obvious difference in the relaxation curves from each cohort, although day 1 samples have a slightly higher deviation from the values of the control group. Overall, there is a relaxation of approximately 60 % from the peak strain of each sample.

**Fig. 7:**
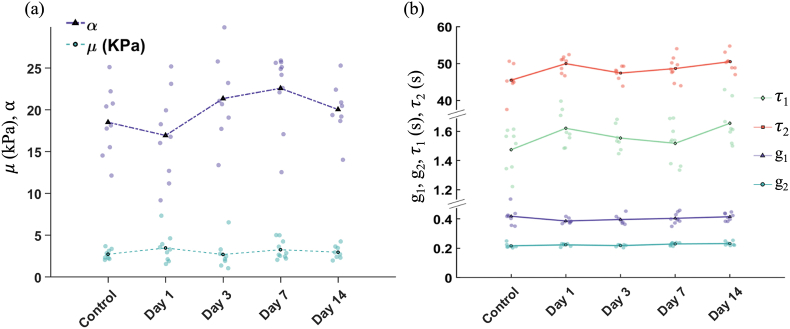
Global elasticity and viscoelasticity parameters. (a) Ogden coefficients (μ and α) and (b) Prony coefficients, are plotted for wounds at days 1–14 and control skin.

The global parameters of elasticity μ (MPa) and strain hardening coefficient, α (dimensionless), and viscoelasticity, g_1_-_2_, τ_1_-_2_ (sec.) were obtained by fitting the stretch-stress curves from tensile testing with Eqs. (1), (2), and results are plotted in Fig. 7a and b respectively (mean and standard deviations can be found in **Sup**. **material Section 6**).

Despite day 7 showing a steeper stress-strain curve on average, such difference is also not statistically significant when looking at the Ogden coefficients. Day 1 displays a slight decrease in the α values, but again this difference is not significant.

Similarly for the viscoelastic parameters, no significant changes can be observed between different wounds in any of the coefficients.

### 3.4 Wound deformation: an overview of the local changes

**Fig. 8:**
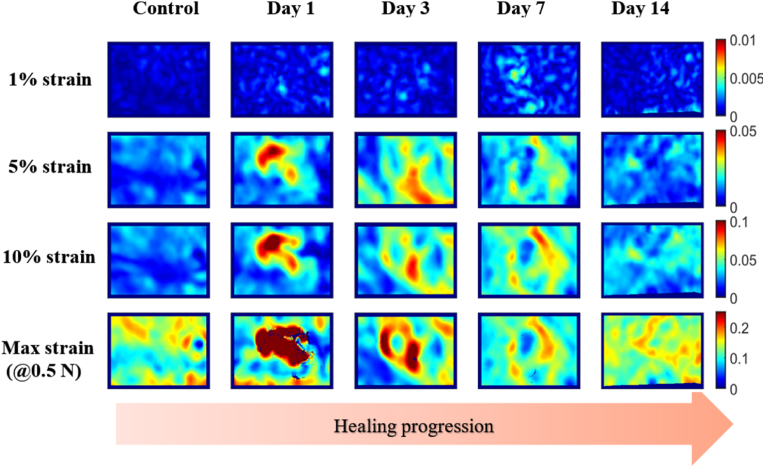
Strain maps showing Green-Lagrange equivalent plastic strains at various levels of sample extension and throughout each healing phase. Within each heatmap, higher strains, and therefore lower strength, are shown in red, equivalently, higher strength material is shown in blue. The strain distributions show certain repeating patterns that correlate to the wound stage. The higher the stretch the clearer the differences between days. (For interpretation of the references to colour in this figure legend, the reader is referred to the Web version of this article.)

Typical examples of strain maps obtained at each different stretch and healing time point are shown in Fig. 8. The colour scale of each strain level is adjusted to the range of values to which the samples are subjected to (i.e., 0–0.01 % for 1 % strains, 0–0.05 % for 5 % and so on). The represented strain corresponds to the Von Mises equivalent strain, which was obtained with Eq. (3).

Clear deformation gradients can be seen in the central area of the wounded samples (*i.e.,* within and around the injury), which dissipate (and practically disappear) when the wound is closed and the scab has fallen (by day 14). Day 1 wounds display higher deformations at the wound core (especially noticeable at the highest stretches), but as the healing progresses, the core seemingly becomes gradually stiffer and the area around the wound becomes concurrently more compliant in comparison.

### 3.5 Regional differences: quantification and correlation to collagen distribution patterns

**Fig. 9:**
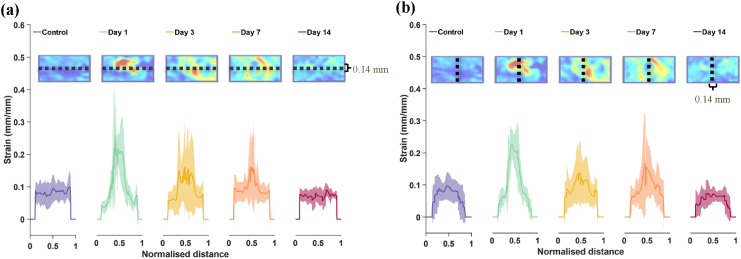
Averaged strains taken along a horizontal (a) and vertical (b) section through the skin under 10 % stretch loading at different wound days. Dashed lines indicate section locations (a 0.14 mm width selection in each). Data is shown mean (solid line) ± SD (shaded regions), for each day.

In Fig. 9, the strains over the stripes described in Fig. 2 (here represented with a black dashed line in all the miniature images on the top) are represented for each day. For ease of visualisation, samples are shown at 10 % stretch.

In day 1 wounds, we observe deformations that are higher at the wound core in both the longitudinal and transversal directions, as was seen in the strain maps of Fig. 8. In day 3 and day 7 samples, the wound area is still more deformable than baseline values (i.e., than healthy skin values). However, the differences between the wound core and its surrounding area are less defined. Despite this, there is a progressive narrowing in the peak strain profiles: when measuring the width of the area where higher deformations start and end, this decreases from 50 % of the total length on day 1, to 36 % on day 3, and 18 % on day 7. Stress reduction in the transversal direction is less substantial, but still drops from 46 % to 40 %, and to 30 % on day 1, 3 and 7 respectively. Day 14 samples display a very similar strain distribution to control samples in both longitudinal and transversal directions.

**Fig. 10:**
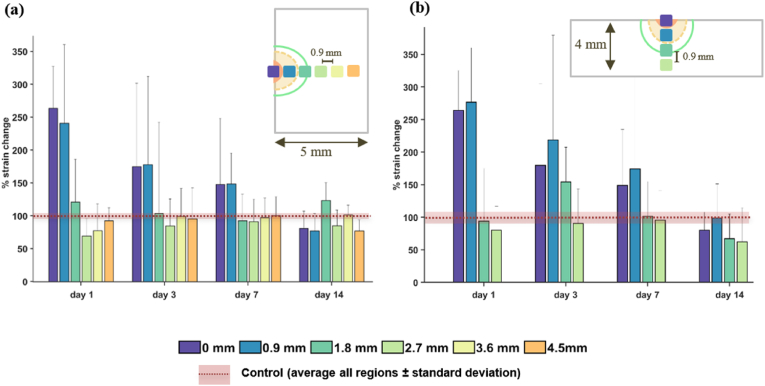
Percentage changes of strain with respect to control group (region to region, at 1.1 stretch). (a) Changes in the longitudinal direction and (b) in the transversal direction. The centres of each sample region are located at 0.9, 1.8, 2.7, 3.6 and 4.5 mm from the centre. The size of the wound is also represented (green line corresponds to day 1, yellow dash line to day 3, and orange area corresponds to day 7 wounds). (For interpretation of the references to colour in this figure legend, the reader is referred to the Web version of this article.)

To study regional differences individually and quantitatively, percentage changes with respect to baseline values at different distances from the sample centre were calculated, as illustrated in Fig. 10.

We observe that the strain at the wound core on day 1 is more than 160 % points higher than its homologous region in a control sample and it stiffens gradually (75 %, 50 % and −20 % points higher). That last value coincides with the 80 % recovery reported in the literature for healed skin [18], although complete healing of the wound cannot be assumed after just 2 weeks. Due to the sample being narrower in the transversal axis, to the cropping performed for DIC analysis (to avoid detection errors in the free boundaries), and due to the Poisson effect during loading, there are fewer points to analyse in that direction. Despite this, the bar profiles evolve in a similar way than those in the longitudinal direction.

In most samples, the regions far away from the centre have closer values to those of healthy skin, indicating that the wound no longer has a measurable impact on the mechanical properties in those regions. Day 14 samples appear to have generally lower values of deformation, but they follow the logical evolution that correlates to their healing status.

## 4 Discussion

Wounds in skin can reduce quality of life, use substantial healthcare resources and if not treated carefully can result in chronic wounds [48]. Characterising wounds quantitatively at different time points is important for the early detection of impairment in the healing process, to provide the most optimal treatment and reduce care times and costs. The current diagnostic methods of chronicity are qualitative and prone to inaccuracies, which often prolongs recovery times and leads to health complications. In this research, our primary goal was to find the link between the different healing phases in wounds, and changes in the mechanical properties of the skin containing them.

### 4.1 Skin's strain-balancing behaviour around wounds

In many previous studies of wound healing mechanics, tensile testing has been the core technique used. However, most mechanical tests are performed to failure as these provide important information regarding the tissue strength and crack propagation patterns. A few works have also analysed wounds using a lower range of forces, but most of them have either been limited to studying the latest stages of healing (commonly, after wound closure or on scars) or have only characterised a few sparse points at the wound core and on its edge.

Despite evident macroscopic differences in wound appearance and sizes, we found no significant differences or clear trends in the global (i.e. full sample) tensile mechanical parameters of elasticity and viscoelasticity measured between wounded and unwounded samples (Figs. 5, Fig. 6, Fig. 7). This was despite a large proportion of the skin sample containing a wound. Remarkably, the skin adapts its localised structure and strain-balancing in order to retain its global/wider load bearing capacity. This has been observed in the tearing of skin [49] but we have now reported it at lower strains and in healing wounds. The local changes observed in this study (Fig. 8, Fig. 9) are likely due to the rapid adaptation of its collagen structure (e.g., Fig. 4) to balance even small loads, without any impact on the bulk tissue mechanical perception. Yang et al. [49] and Pissarenko et al. [50,51] have previously postulated that such strain balancing could be the mechanism responsible for masking global mechanical differences. At our lower strains this balance could assist in avoiding stress concentrations that could damage skin's healing.

Coupling our mechanical observations with global properties, we did observe reduced cross-sections of the skin once wounded, which we expected (per classical mechanics) to have affected the peak stresses measured at same deformation levels, but again this was not detected. Our Ogden model results only showed a slight variation day to day, but the data variability was too large for statistical confidence. For instance, day 1 samples had a lower strain hardening component compared to other time intervals, which could be due to the reduced amount of collagen overall in that cohort (due to the presence of a 4 mm hole), as this protein is the main component responsible of the J-shape behaviour when tensioning soft biological materials. On the other hand, day 7 samples presented a steeper curve which can be indicative of an increase in stiffness, presumably due to the presence of scabs and wound contraction at that stage. However, the variation in the data is too big to confidently associate these results to such biological features.

Considering the viscoelastic effect on skin, generally, higher long-term relaxation coefficients have been associated with a higher hydration on the skin layers [52]. We would therefore anticipate that viscoelastic parameters in wounding would be related to the presence of exudate, higher cellular content in early-stage wounds, increased blood flow, or to the density of the collagenous networks. In practice, no viscoelastic changes were found amongst our timepoints (Fig. 6): the average relaxation curves for all groups were, again, practically identical at each stage. The Prony model was used to quantify the statistical differences between the relaxation coefficients extracted from them, but no significance was found. Overall, global measurements were not sufficient to aid in wound staging.

### 4.2 Local strain observations

The strain maps from Fig. 8, revealed obvious differences in the deformation patterns from each timepoint of healing, which were observable even at very low strains (5 %). On average, the maximum strains were experienced by the wound core of day 1 samples. As healing progresses, the core seems to become stiffer, while the surrounding area becomes more compliant (as noted above). In attempting to quantify these progressive changes across and along a wound, we selected small rectangular areas (0.6 × 0.14 mm) at fixed distances from the centre of the sample (in particular: at 0.9, 1.8, 2.7, 3.6 and 4.5 mm). We found that the wound core deformation on day 1 was 160 % points higher on average than that same region in the control group, and reduced to more than half that value (75 % points higher) by day 3, and by a third (to 50 % points higher) by day 7. By day 14, the core appeared to be less deformable than the analogous baseline region (−20 % points), which agrees with the mechanical values reported in the literature for tissues recovered after an injury [18].

Using the discrete approach depicted in Fig. 2, we did not find significant differences between the wound core and the surrounding area, which contradicts the results from Pensalfini et al. However, our process used points at fixed tissue positions, and the wound centre selection was performed by looking at the wound images (Fig. 2a) instead of at the strain maps (Fig. 2c). Thus, our discrete points were generally not as well placed to find the boundaries between different regions, as these were restricted to a straight line that could have diverged from the major loading axis. We also note that our selection of points may have not been sufficiently dense to have identified the optimal sensing positions. Nevertheless, local measurements (i.e., Figs. 8, Fig. 9, Fig. 10) are still useful when developing future wound assessment technologies, as day-to-day differences were clearly perceived in the deformation maps and in the strain curves extracted from them.

### 4.3 Correlation of mechanical changes in wounds and their biostructures

During the early stages of healing, different cells work to generate new tissue in the wound bed. Importantly, fibroblasts lay out collagen which help the wound close: the loosely arranged fibrils they secrete are self-assembled into thicker collagen fibres, whose compaction pulls the surrounding tissues together [53]. This tension results into a higher fibre alignment both within the wound bed and in wound adjacent areas, as was observed in Refs. [21,54] and has now been quantified with a collagen alignment coefficient.

As the healing evolves and the wound is filled (as indicated by an increase in the fibre density of the histological images, which goes from a 61.51 % ± 6.97 fill on day 1, to an 83.99 % ± 5.97 fill by day 14), the collagen networks start to unpack and lose tension (i.e., during the remodelling phase). This results in an increase of the polar intensity at more angular positions. Thus, the alignment values are significantly higher on day 1 (p < 0.005), and gradually decrease towards the baseline (or control) values by day 14.

The local strains experienced by each timepoint follow the same trends than those of the fibre alignment coefficients shown in Fig. 4 (see **Sup**. **Materials** section 5). Thus, a correlation between a healing degree feature (i.e., collagen network alignment) and biomechanical properties (i.e., local strain) has been corroborated with this study. This has relevance for those seeking to understand how the internal structure of tissue may be inferred by minimally invasive measurements. In particular, as the strains used were physiologically relevant, we could envisage the use of small strain measurements in wounds to assess their healing, using either some form of wearable or non-invasive visual assessment for patients. Importantly, identifying wounds that were not healing well could then lead to a substantial benefit to both patients and healthcare systems.

### 4.4 Wound mechanics as a measure of healing – study limitations and clinical potential

Beyond the study limitations detailed previously, skin's anisotropy and location-dependency material properties may mean that the healing timescales or strain distributions can differ from wound to wound [55,56]. Multiaxial skin testing may provide more detail here, although we believe that single-orientation strains are likely to be more representative of local tissue strains in-vivo. Regardless, we would expect similar trends to those shown in Fig. 8, Fig. 9, Fig. 10, that is: an initial increase in the deformation experienced by a large area within the wound followed by a gradual decrease (of both the deformation and the area) in time, reaching baseline values as the healing concludes.

Despite this, we note that the data obtained using the Ogden models showed substantial intra sample variability. Thus, the use of an analytical model may not have been ideal, and in the future, using data-driven models (such as the Bayesian approach of Aggarwal et al. [58]), could provide greater independence from these intra-group variations.

It is also known that skin's properties can be affected by both environmental and intrinsic factors (e.g., by the hydration levels of the individual being tested, temperature, etc.) [33,57]; however, analysing daily variations of one same wound belonging to the same subject over time has not been possible due to the *ex vivo* nature of this study. These variations may affect the quantification of strain in absolute value terms, but we would expect that measurements of different surface locations relative to each other would still show an evolution (or lack thereof) of the healing, as stated in the previous paragraph and seen in Fig. 8, Fig. 9, Fig. 10.

Thus, whilst the work within this paper relates to a pre-clinical mouse study, and the methods have only been applied to excised skin of healthy subjects, the ability to assess skin healing using mechanics at physiologically relevant levels demonstrates opportunities for future non-invasive, quantitative technologies. Some differences are to be expected in the healing mechanisms between humans and mice, and between healthy and unhealthy subjects, which should be further investigated with the implementation of more advanced wound healing models (out of the scope of the present study). Nevertheless, we believe that the present study provides a crucial baseline that can be referred to in ongoing and future biomechanical work. Our findings show that local measurements on the skin's surface are able to provide quantitative insights into biological behaviour that goes undetected when using non-invasive/qualitative clinical methods, as well as when using bulk measurements. Refining the present approach, will provide a strong path towards the expansion in the field of ‘smart’ bandages and wearable technologies.

## 5 Conclusion

Wounds cause a decrease in quality of life and consume large amounts of healthcare resources, largely due to their management, which is still subjective and based on observation. In this study we sought to quantify how wounds at different healing stages change their mechanical properties and how these could be measured at clinically relevant strain levels, as a step towards quantitative wound tracking technologies. We undertook this in mice and found that the inherent protective mechanisms of skin (e.g., the biostructural arrangement of the collagen fibres and the ability to balance load), demonstrated a remarkable ability to mask the effects of wounds across a full skin sample. No significant differences were found in the stress-strain curves of tensile testing, or in the viscoelastic parameters obtained through relaxation experiments when comparing wounded and unwounded skin tissues in bulk.

However, when studying mechanical deformations locally using digital image correlation, we found that the surface strain patterns were varied substantially depending on the healing stage. For instance, in day 1 wounds high strains were present within the wound, but this changed at day 3 onwards where skin surrounding the wound became more compliant to take the major strain impact. This surface-visualisation of strains provides important insights towards the future of wound management.

Studying early acute responses versus late (and stabilised) ones is crucial to develop diagnostic tools that can objectively quantify and distinguish different points on the healing spectrum. Adaptations of these approaches to clinical use have the potential to identify early indications of wound healing problems such as excessive inflammation or chronicity.

## Statement of significance

In this study, we have characterised both the mechanical response of wounds in the early stages of healing and their biostructural arrangement. We have summarised each feature by generating simple-to-interpret quantitative indexes: a collagen alignment value and normalised percentage change in local strains at fixed locations based on the tissue's baseline (i.e. unwounded) values under normal physiological strains. These data can be used to inform the design of new wound healing diagnostic tools that use the mechanical properties of the tissue as a health biomarker (e.g., wearable sensors).

## CRediT authorship contribution statement

**Sara Medina-Lombardero:** Conceptualization, Data curation, Formal analysis, Investigation, Methodology, Visualization, Writing - original draft, Writing - review & editing. **Connor Bain:** Data curation, Formal analysis, Investigation, Methodology, Software, Visualization, Writing - original draft, Writing - review & editing. **Laura Charlton:** Investigation, Methodology, Visualization. **Antonella Pellicoro:** Investigation, Methodology, Resources, Visualization. **Holly Rocliffe:** Investigation, Methodology, Resources, Visualization. **Jenna Cash:** Conceptualization, Formal analysis, Funding acquisition, Methodology, Supervision, Validation, Writing - original draft, Writing - review & editing. **Robert Reuben:** Formal analysis, Methodology, Supervision. **Michael L. Crichton:** Conceptualization, Formal analysis, Funding acquisition, Methodology, Project administration, Resources, Supervision, Validation.

## Declaration of competing interest

The authors declare the following financial interests/personal relationships which may be considered as potential competing interests:Michael Crichton reports financial support was provided by 10.13039/501100000266Engineering and Physical Sciences Research Council. Jenna Cash reports financial support was provided by 10.13039/100010269Wellcome Trust.

## Data Availability

Data will be made available on request.
