# Supplementary material for: The biomechanics of wounds at physiologically relevant levels: Understanding skin's stress-shielding effect for the quantitative assessment of healing
Source: Mater Today Bio. 2024 Jan 17;25:100963. doi: 10.1016/j.mtbio.2024.100963 (PMC10835282; doi:10.1016/j.mtbio.2024.100963)
Supplement: Multimedia component 1 [file mmc1.docx]

**SUPPLEMENTARY MATERIALS**

1. **Wounds position, sample size and marking process pre-excision**

To account for the contraction of skin, a stencil was used to mark the lines that would compose the final dimensions of the samples for the tensile tests. These dimensions are: 40 mm long, 10 mm width, and a gauge length of 20 mm. A hole in the stencil was used to ensure that the wound remained in the central area of the sample. This design allowed the surface of the tissue to remail free from other markings to aid DIC measurements.

**
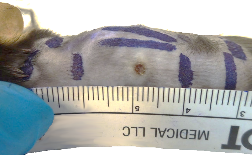

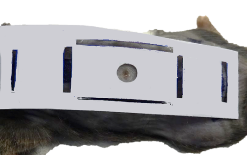
**


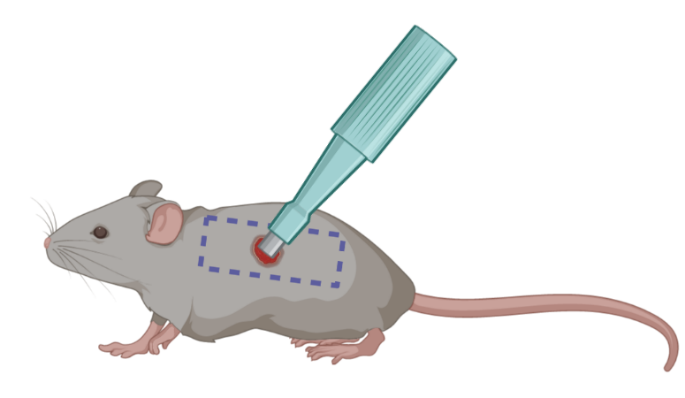


Punch biopsy (x2)
ø 4 mm

**(a)**

**(b)**

**(c)**

20 mm

10 mm

10 mm

10 mm

Tweezers to aid with the paper removal

Pipetted water

**(d)**

**
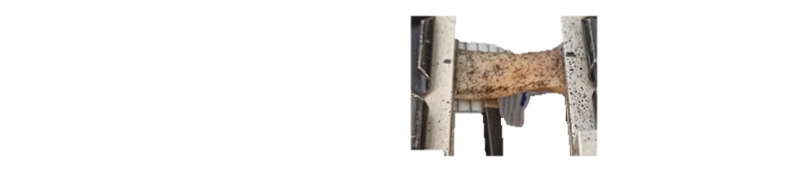

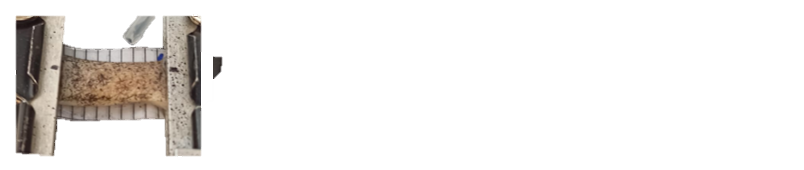
**

Sacrificial paper

***Fig. S1. Illustration showing the location and dimensions of the biopsy wounds and the specimen, and the process of marking and mounting the samples on the equipment.*** *(a)* *Location of the dorsal wound and direction of the excised specimen within the animal. (b) Stencil used to mark the sample prior to excision. A hole allowed to see the wound (when present) and place it in the centre of the specimen to test. (c) Sample dimensions. (d) To transfer the sample from the preparation bench to the tensile testing equipment, a sacrificial paper was used, which once the sample was in place was removed with water and flat tweezers, as depicted.*

1. **Speckle size, ensuring goodness of measurements**

The sizes of the speckles used for tracking the local deformations were measured on 20 skin samples. Median sizes achieved were of 8.19 ± 0.76 px (approx. 160 µm pixel size), and covered a 44.88 ± 4.89 % of the sample’s surface on average, as measured with CellProfiler (v4.1.3), see Fig. S2.


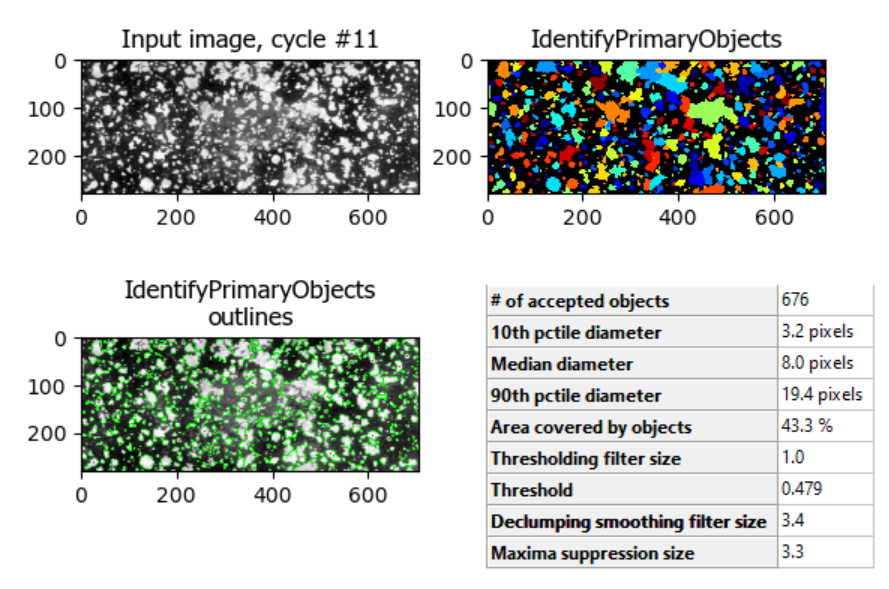

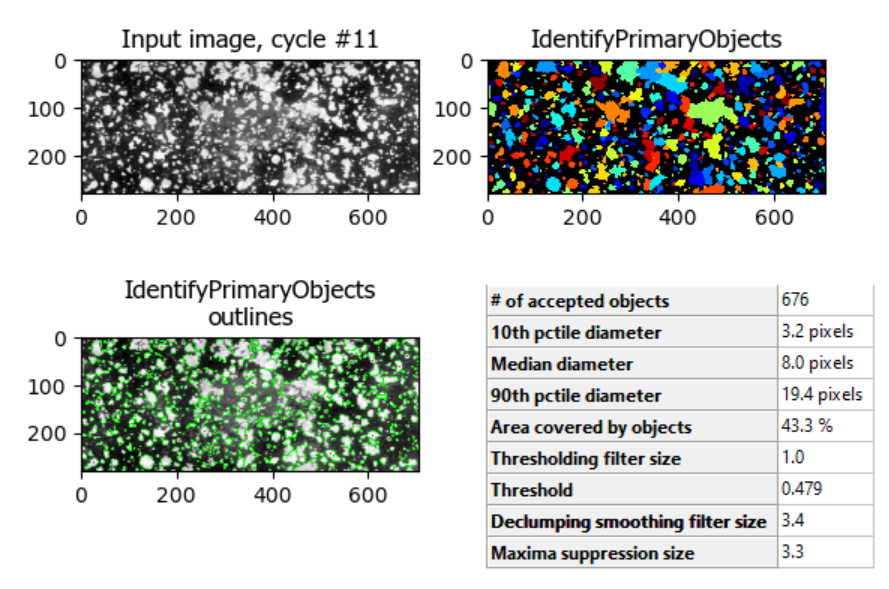

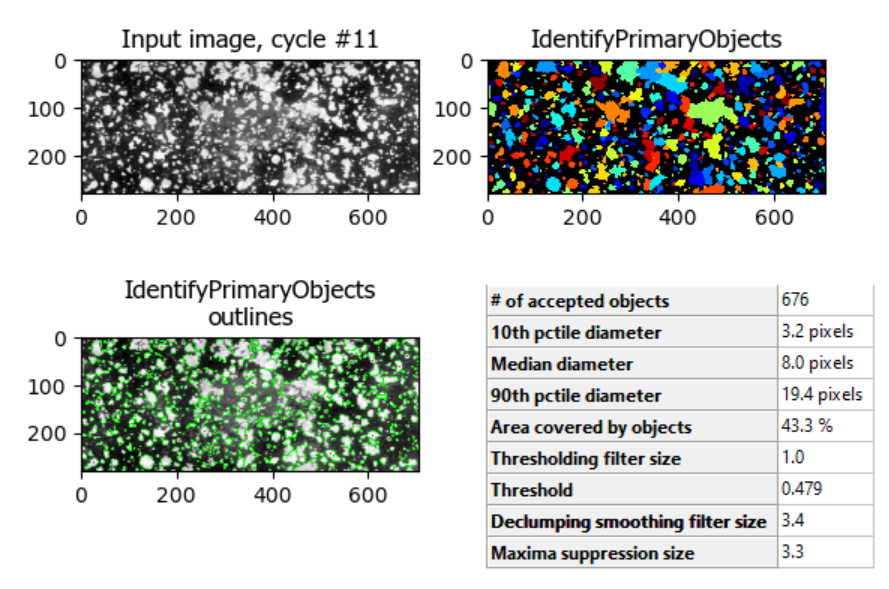


**(a)**

**(b)**

**(c)**

***Fig. S2.*** ***Example of the processing in CellProfiler to acquire the density and size of the speckles****. (a) Shows the original cropped image after applying a series of filters to digitally enhance the pattern. (b) The contours of the speckles are detected. (c) The speckles are classified according to their size using different colours.*

Craig [1] recommended using patterns that covered approx. a 50% of the sample with speckle sizes of 3-5 pixels (on non-biological materials), whilst Mallet [2] proposed using 100 µm pixel size, although only achieving sizes of 152 ± 9 µm in the referenced study, which was still considered optimal, due to the expected experimental difficulties when using biological specimens. Thus, the pattern employed in the present work (~160 µm pixel size) was found sufficient for detecting strains across the whole surface of the samples, although it is recognised that this can be further improved to allow local studies with higher resolutions.

Whilst water efflux is generally normal in biological samples, none was observed during the loading of mice skin samples (this loading was generally less than 6 seconds long). Thus the pattern on the surface of the samples was generally unaffected by this phenomena (see **Fig. S3**).


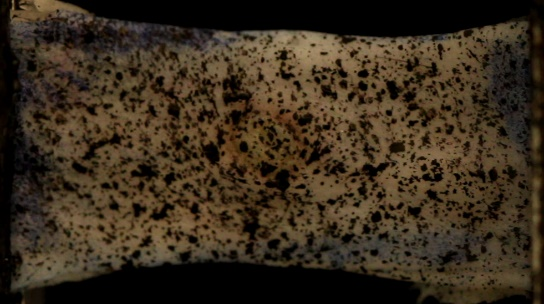

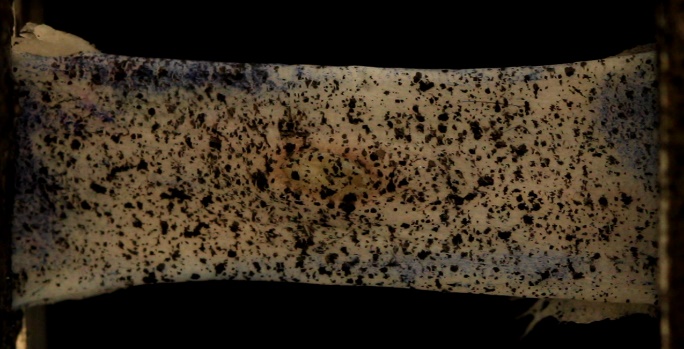


**(b)**

**(a)**

***Fig. S3. Patterned wounded sample before (a) and after loading a sample to full stretch (~0.5 N) (b).*** *No glares can be observed in any of the video frames,* *which would correspond to water (or grease) on the sample’s surface.*

The accuracy of the DIC experimental approach was also assessed by performing tensile experiments on homogeneous PDMS samples of known material properties and using the experimental test data to compare the strain maps to those generated with a computer simulated model. A good agreement was seen when comparing the trends of the real vs the simulated tensile test data, as observed in **Fig. S4** (offsets were attributed to various reasons, such as: differences of the actual material parameters to those encountered in the literature, small calibration errors, etc.).


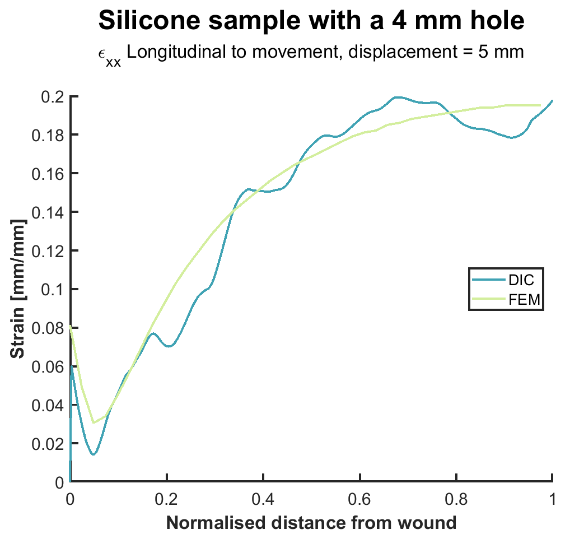

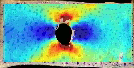

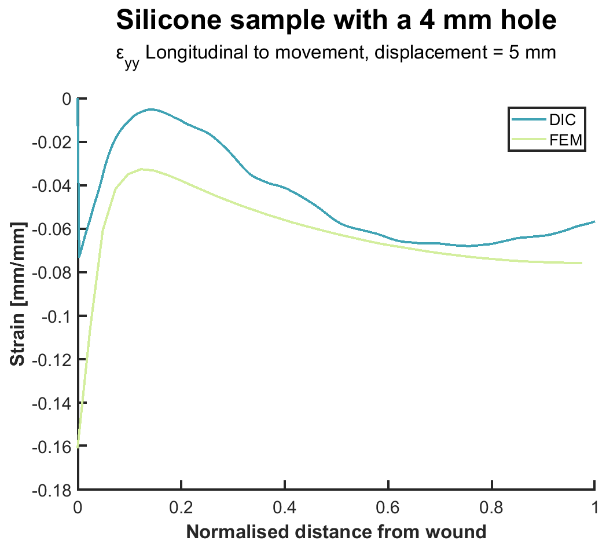

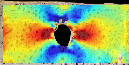

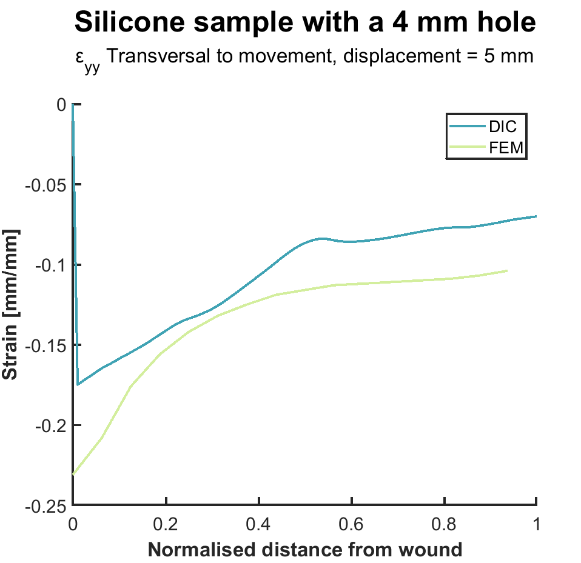

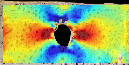

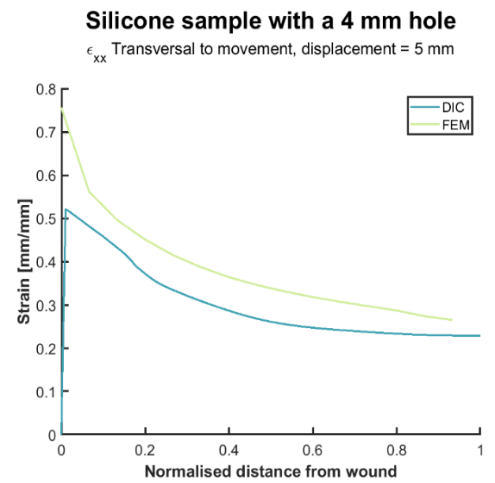

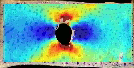


***Fig. S4. Comparisons between DIC results and FEM of a ‘wounded’ PDMS sample.*** *(a) and (b) correspond to in-plane strain ε_xx_, (c) and (d) to ε_yy_ . The directions of each line measured (longitudinal or transversal) are indicated in the miniature image with a dashed line, as well as in the titles of each figure*

Despite the simplicity of the computer model (i.e., 2D deformable shell, the parameters of which can be shared upon request) and homogeneity of the PDMS samples (not representative of the conditions usually found in skin), this test was useful to determine whether out-of-plane movements (which could result in a defocus of the camera) would affect the surface measurements throughout the testing, as well as to evaluate the performance of the selected software and camera system.

1. **Fixation and staining protocols for the histologic analysis**

For fixing the sectioned samples, the embedding cryomatrix was first removed with a series of dilutions (100% - 80% - 40% EtOH, 5 minutes each, followed by 3-4 dips in water). Note that this part of the protocol was adapted following expert advice encountered in an online forum (<https://www.researchgate.net/post/Picrosirius-overstaining-in-frozen-muscle>), to avoid overstaining issues. The sections were then submerged in a fixative solution (Formal-fixx 1:4 dH20) for half an hour. Once fixed, samples were left in PBS until staining to prevent their dehydration.

For the collagen staining, the tissue was submerged in the PSR solution (0.5 g Sirius Red, 1.3% picric acid in dH2O, Sigma-Aldrich) for an hour. Afterwards, acid water (5 ml acetic acid (glaciar) in 1 litre of water) was used to wash off the excess PSR and after gentle shake drying, the samples were dehydrated with 3 changes of 100% EtOH. Lastly, xylene was used to clear the samples (two immersions of 30 seconds) and to finalise, they were mounted with DPX (Sigma-Aldrich) and a coverslip overnight. With PSR staining, collagen appears in pink-red under brightfield microscopy with a pale yellow background [3]–[5].

1. **FIBRAL working principles**

FIBRAL is a bespoke MATLAB tool designed to quantify the alignment of fibrous macrostructures in histological slides. Using two primary algorithms (colour-based segmentation and Fourier-based analysis), histology images are analysed to calculate a global directionality quotient. To fully isolate collagen fibrils from non-fibrous tissue, FIBRAL initially performs colour-based segmentation. The user selected image is converted from an RGB image to a 2-dimensional matrix of form $m$ x $n$ x 3 where $m$ and $n$ represent the row and column indices of the three distinct colour channels.

Note that the resolution of the images provided in **Fig. 4 (a)** in the main body of this paper is lower than in the original files. In **Fig. S4 (b)** a zoomed in section has been added to show the original image resolution that was used for the analysis.

**(a)**

**(b)**


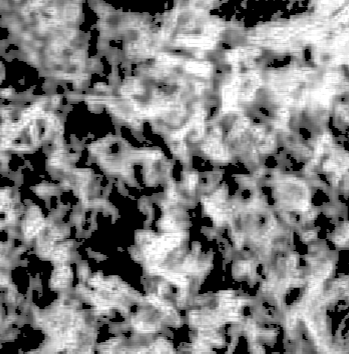


**(c)**


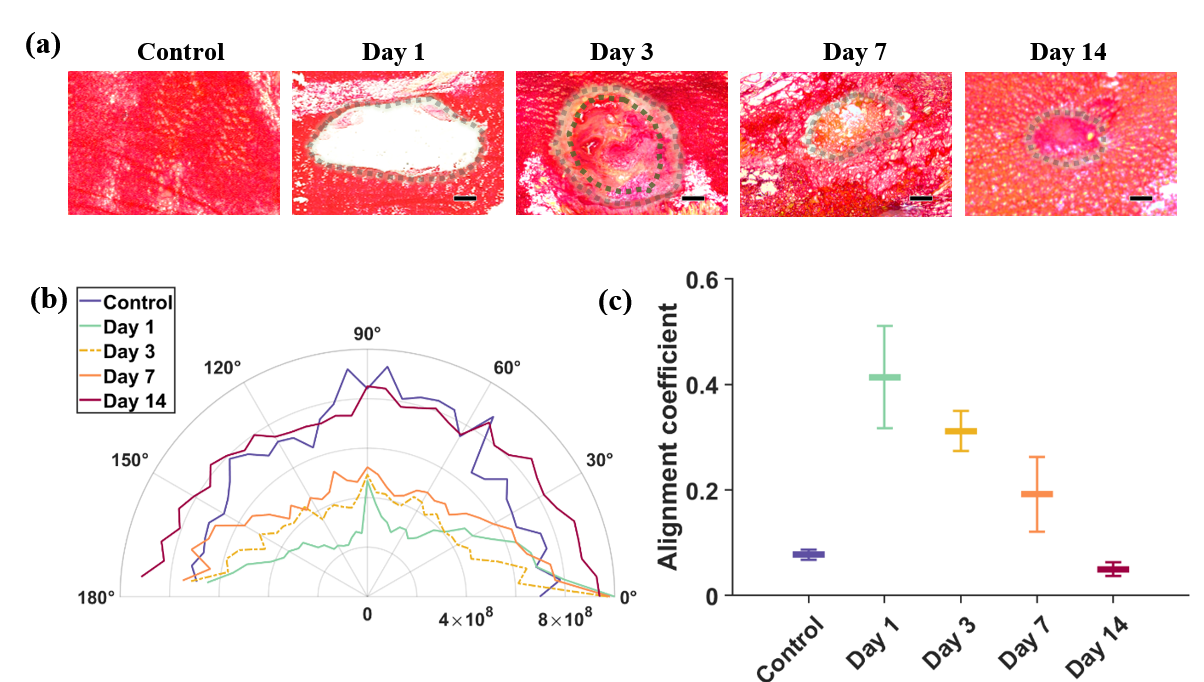

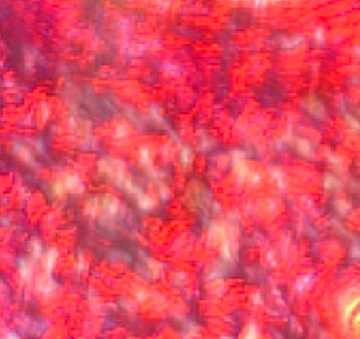


***Fig. S5. Representative example of a zoomed in histologic image to demonstrate resolution of the original datasets used for the analysis. (a)*** *Full image of a PSR-stained 14 day skin wound, in low resolution.* ***(b)*** *Small section of the same image at higher resolution, in which fibres can be appreciated with the naked eye (in bright red).* ***(c)*** *Same high-resolution image after initial processing. Fibres are now shown in a grayscale and background colours have been reduced.*

The RGB image (**Fig. S5b**) is converted to the L*a*b colour space where we isolate the a-channel and the positive region of the b-channel. To the resultant greyscale image, we increase the contrast and brightness by 20% and 5% respectively (**Fig. S5c**).

Upon completion of the colour segmentation steps, the preconditioned image is converted to the spectral domain using a 2-dimentional fast Fourier Transformation. A frequency shift was performed to centrally contain low-frequency components, with higher frequencies distributed radially towards the outer edges of the image. To determine the angular distribution of frequencies we convert from Cartesian to polar coordinates, resulting in a 2-dimensional image whereby all pixel values contain within a single column index represent all Fourier frequencies at an equal angular bin on the base image (i.e. the pixels of column 5 represent the frequencies of the base image at 5 degrees to the horizontal central axis). Large pixel intensity values within a single column represent the summation of multiple high amplitude sinusoids; signifying major fibre alignment along the respective orientation.

To generate a quantifiable metric which describes the level of anisotropy present in a collagen fibre matrix a two-dimensional array is created which stores the overall intensity value at each one-degree bin size:

$$\theta_{ij}=\left[ \begin{matrix} \begin{matrix} \alpha_{1} & \alpha_{2} \end{matrix} & \begin{matrix} \ldots& \alpha_{180} \end{matrix} \\ \begin{matrix} I_{1} & I_{2} \end{matrix} & \begin{matrix} \ldots& I_{180} \end{matrix} \end{matrix} \right]$$

Where $\alpha$ and $I$ relate to the angle and intensity values at each 1 degree bin size. To analyse the directionality of the fibre distribution, we create a direction vector at each bin with a magnitude equivalent to the summed intensity value of the pixels:

$$\left[ \begin{matrix} x \\ y \end{matrix} \right]=\left\{ \begin{matrix} I_{ij}\times\cos\alpha\\ I_{ij}\times\sin\alpha\end{matrix} \right\}$$

From this, the dot product is calculated using ach permutation of two vectors:

$$d_{ij}=\frac{v_{i}.v_{j}}{\left| v_{i} \right|\left| v_{j} \right|}$$

Parallel vectors produce a dot product of 1, with orthogonal vectors producing a dot product value of -1. All results are then normalised such that they lie between 0 and 1. All $N$ segments can then be averaged to give an overall directionality quotient using:

$$d=\frac{\sum_{i=1}^{i=N} \frac{\sum_{j=1}^{j=N}}{N}}{N}$$

A directionality quotient of 1 represents an ideally linear case, where all fibres are oriented along one angle, whereas a directionality quotient of 0 represents ideal isotropy where an equal proportion of fibres are oriented across all angles.

The code has been benchmarked against the widely used Orientation-J plugin from ImageJ (Fiji). Whilst the results obtained were similar in most cases, Fibral performed better in dense or low-contrast sections, as it would detect and isolate individual fibres even in those scenarios, rather than clumping these sections as if they were single elliptical fibres.

1. **Correlation between biostructural and biomechanical features**

A linear regression was performed to quantitatively assess the relationship between the average collagen alignment of each cohort and biomechanical performance of the tissues, as measured in the centre of the wound (see **Fig. S6**).


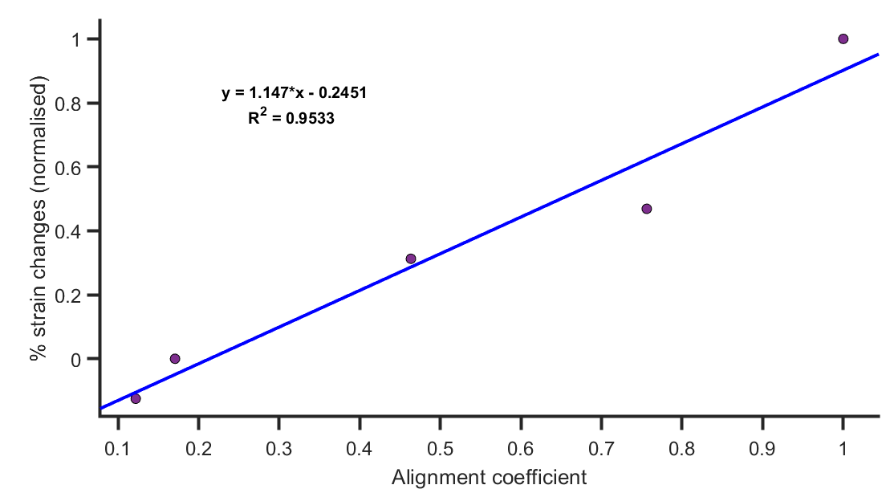


***Fig. S6. Linear regression fit to analyse the correlation between the overall alignment coefficient (normalised) and percentage biomechanical changes (i.e. strain) in the central area of the wound.***

The slope of 1.147 indicates that as the collagen alignment increases, there is a concurrent change of a similar magnitude in the biomechanical properties. The r-square value of 0.95 suggests a strong relationship between the two.

On the other hand, when comparing the alignment coefficient vs time (**Fig. S7**), it can be observed that the data follows an exponential decay.


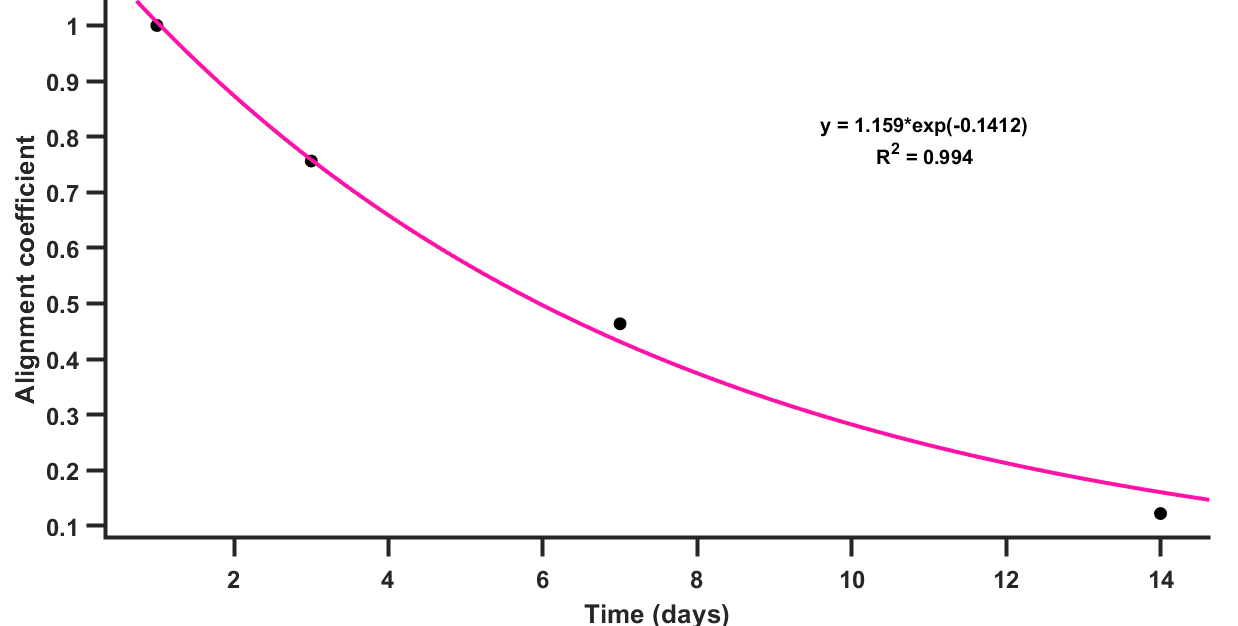


***Fig. S7. Exponential regression fit to analyse the correlation between the overall alignment coefficient (normalised) and wound age (in days).***

As time progresses the alignment decreases and tends towards values corresponding to those of healthy skin. Thus, the behaviour displayed in **Fig. S7** captures the expected wound healing trend over time, with a strong initial response (inflammation phase) followed by a gradual fibre remodelling over time.

1. **Statistical analysis**
2. **Alignment coefficients**

A t-test was carried out (with a post-hoc Bonferroni correction), to evaluate the significance of the differences between groups shown in **Fig. 4c**. The p-values between groups are tabulated below.

**Table S1. P-values when comparing alignment coefficients of the different cohorts** (c – control, d1 – day 1 wounds, d3 – day 3 wounds, d7 – day 7 wounds and d14 – day 14 wounds)

|  | **c** | **d1** | **d3** | **d7** | **d14** |
| --- | --- | --- | --- | --- | --- |
| **c** | 0 | **0.0039** | **0.0005** | 0.0496 | 0.0437 |
| **d1** | 0 | 0 | 0.1656 | 0.0328 | **0.0029** |
| **d3** | 0 | 0 | 0 | 0.0611 | **0.0003** |
| **d7** | 0 | 0 | 0 | 0 | 0.0264 |
| **d14** | 0 | 0 | 0 | 0 | 0 |

**Power analysis:** considering the means and standard deviations obtained for each group, a power calculation was performed in G*Power (v.3.1.9.7) [6] to estimate which sample size would give more confidence to these results. Using day 1 vs control, the minimum number of samples needed was calculated as 3, which already coincided with our group size. Note that for the differences to be considered significant the p-value cutoff was also lowered (p<0.005 instead of <0.05).

1. **Ogden and Prony coefficients (global data)**

The mean and standard deviation values corresponding to Fig. 7 are tabulated below. Note that no significant differences were found between any of the groups (all p-values > 0.05).

**Table S2. Average and standard deviations of the Ogden and Prony coefficients for all wound cohorts**

|  | **µ (kPa)** | **⍺** | **g_1_** | **g_2_** | **τ_1_ (s)** | **τ_2_ (s)** |
| --- | --- | --- | --- | --- | --- | --- |
| **c** | 2.73±0.57 | 18.51±4.05 | 0.42±0.05 | 0.22±0.01 | 1.47±0.15 | 45.49±3.97 |
| **d1** | 3.48±1.75 | 16.93±5.35 | 0.38±0.02 | 0.22±0.01 | 1.62±0.12 | 49.96±2.12 |
| **d3** | 2.71±1.71 | 21.35±5.04 | 0.39±0.03 | 0.21±0.01 | 1.55±0.08 | 47.42±1.93 |
| **d7** | 3.28±1.12 | 22.57±4.41 | 0.40±0.03 | 0.23±0.01 | 1.51±0.13 | 48.65±3.16 |
| **d14** | 2.98±0.77 | 20.04±3.23 | 0.42±0.02 | 0.23±0.01 | 1.65±0.14 | 50.51±2.46 |

1. **Regional differences obtained through strain maps (local data)**

All possible pairwise combinations were tested (i.e., all days vs all days, all regions vs the other regions on the same day). In **Tables S3 & S4** the median and standard deviation values of each cohort and region are shown, and p-values < 0.05 are highlighted.


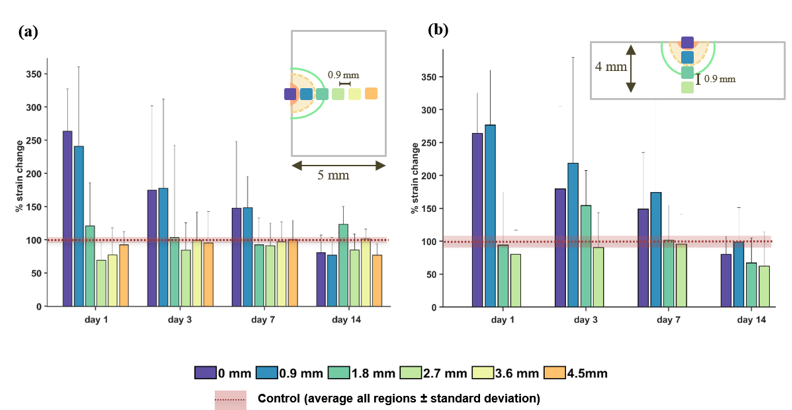
**Table S3. Median and standard deviations of the raw strains extracted at different distances from the sample centre (see Fig. 8 and Fig. 10) in the longitudinal direction.**

|  | **centre** | **0.9 mm** | **1.8 mm** | **2.7 mm** | **3.6 mm** | **4.5 mm** |
| --- | --- | --- | --- | --- | --- | --- |
| **c** | *0.09 ± 0.04* | *0.08 ± 0.02* | *0.08 ± 0.03* | *0.08 ± 0.02* | *0.07 ± 0.03* | *0.08 ± 0.03* |
| **d1** | **0.22* ± 0.05** | **0.19* ± 0.09** | 0.09 ± 0.05 | *0.06 ± 0.02* | *0.06 ± 0.03* | *0.08 ± 0.02* |
| **d3** | 0.15 ± 0.08 | 0.15 ± 0.10 | 0.09 ± 0.10 | *0.07 ± 0.02* | *0.08 ± 0.02* | *0.08 ± 0.03* |
| **d7** | 0.13 ± 0.09 | 0.11 ± 0.03 | 0.07 ± 0.03 | *0.07 ± 0.03* | *0.07 ± 0.02* | *0.08 ± 0.02* |
| **d14** | *0.07 ± 0.02* | *0.07 ± 0.02* | 0.09 ± 0.02 | *0.07 ± 0.02* | *0.07 ± 0.01* | *0.06 ± 0.01* |

p-values <0.05 (after post-hoc corrections) are shown in bold. Baseline values (control and “far-field” regions) are in italics.

**Table S4. Median and standard deviations of the raw strains extracted at different distances from the sample centre (see Fig. 8 and Fig. 10) in the transversal direction.**

|  | **centre** | **0.9 mm** | **1.8 mm** | **2.7 mm** |  |  |
| --- | --- | --- | --- | --- | --- | --- |
| **c** | *0.09 ± 0.04* | *0.07 ± 0.02* | *0.08 ± 0.03* | *0.08 ± 0.05* |  |  |
| **d1** | **0.22* ± 0.05** | **0.18* ± 0.05** | *0.07 ± 0.06* | *0.06 ± 0.03* |  |  |
| **d3** | 0.15 ± 0.08 | 0.15 ± 0.10 | *0.11 ± 0.04* | *0.08 ± 0.04* |  |  |
| **d7** | 0.13 ± 0.07 | 0.12 ± 0.10 | *0.08 ± 0.04* | *0.07 ± 0.03* |  |  |
| **d14** | *0.07 ± 0.02* | *0.07 ± 0.03* | *0.05 ± 0.03* | *0.05 ± 0.04* |  |  |


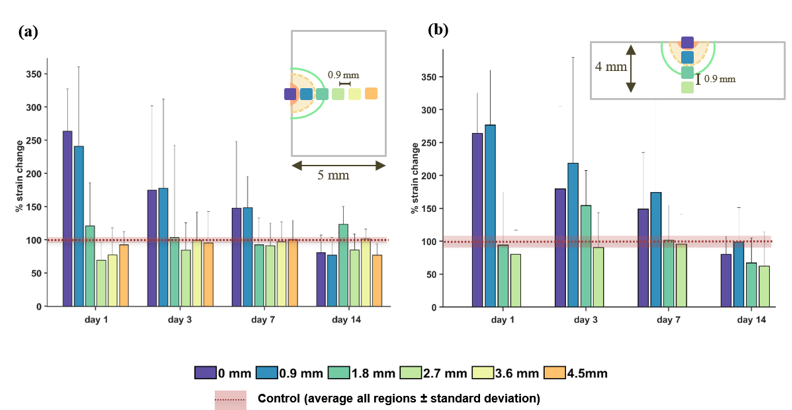


*p-values <0.05 (after post-hoc corrections) are in bold. Baseline values (control and “far-field” regions) are in italics.*

Only significant differences between the core of day 1 (centre) and its nearest region (0.9 mm) were found when compared to “baseline” values (i.e., control, day 14 and far-field regions).

1. **Automated point selection method**

The averaged data shown in **Fig. 9** and **Fig. 10** seems to contradict the patterns observed on the individual strain maps from **Fig. 7**. As it has been briefly discussed, this is due to the automatic point selection method used.

To further expand on this, in the following picture (**Fig. S9**), a wider selection of strain maps of more samples is shown. The black dashed lines show the stripes from where the data was automatically extracted (in the longitudinal and transverse directions).


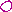

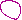

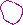

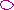

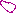

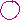

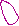

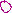

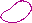

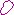


**Control**

**Day 1**

**Day 3**

**Day 7**

**Day 14**

***Fig. S8. Strain maps from different samples on different days.*** *The wound “cores” have been circled (in purple) on days 3 and 7.*

As it can be seen, despite seeing some identifiable features in each day (e.g., day 1 showing the most wound core deformations for all samples, days 3 and 7 showing more deformations around the wound core than within it, and control and day 14 being the most homogeneous), there is still a high variability in each cohort. For instance, some samples have bigger deformation rings around their wounds, some are slightly off-centred or tilted, some are overall less deformed and thus their strain patterns are not as visible, etc. With the automatic point selection, it is not possible to perfectly overlay all the wound areas at the exact same coordinates, which inevitably affected the averages taken for **Fig. 9** and **Fig. 10**.


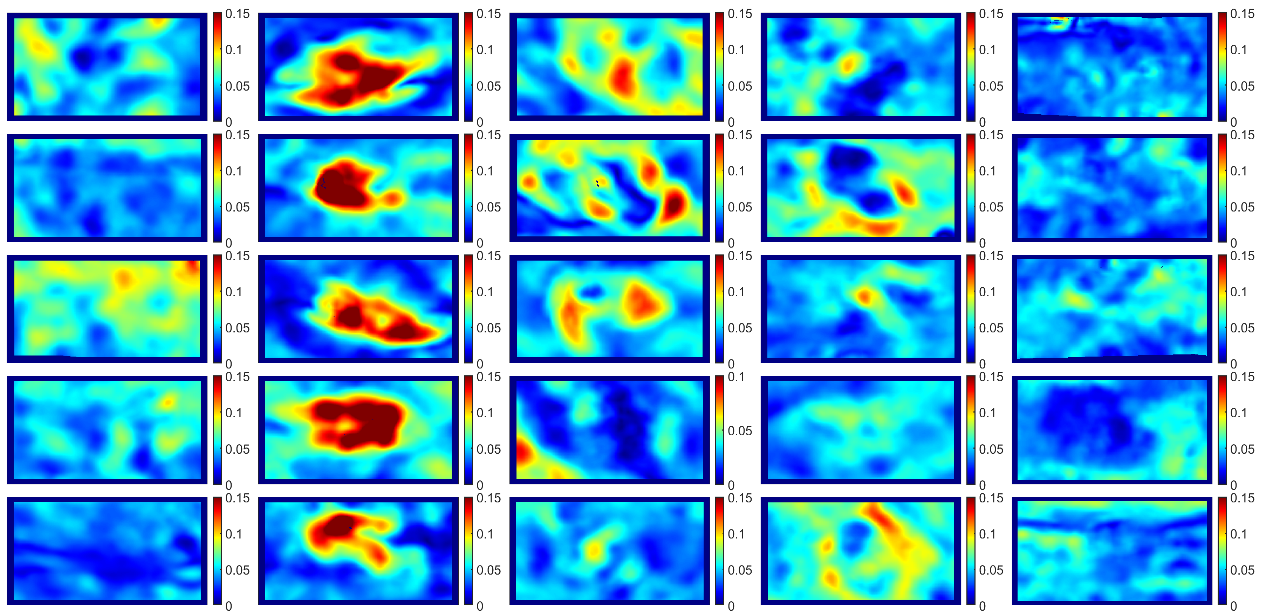

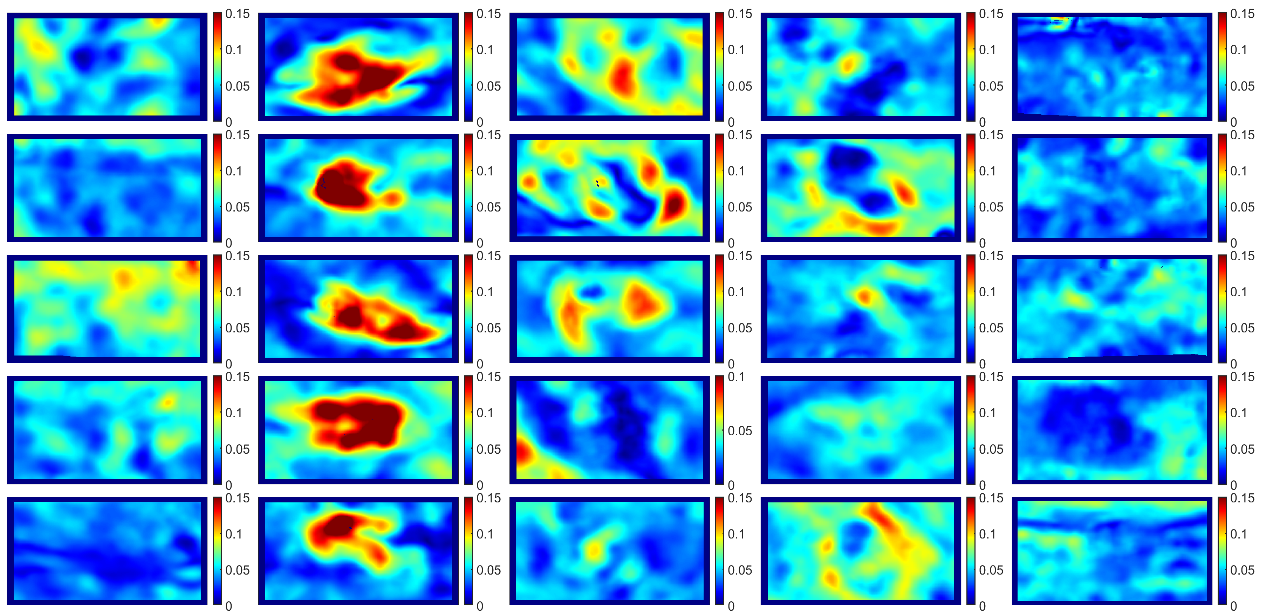

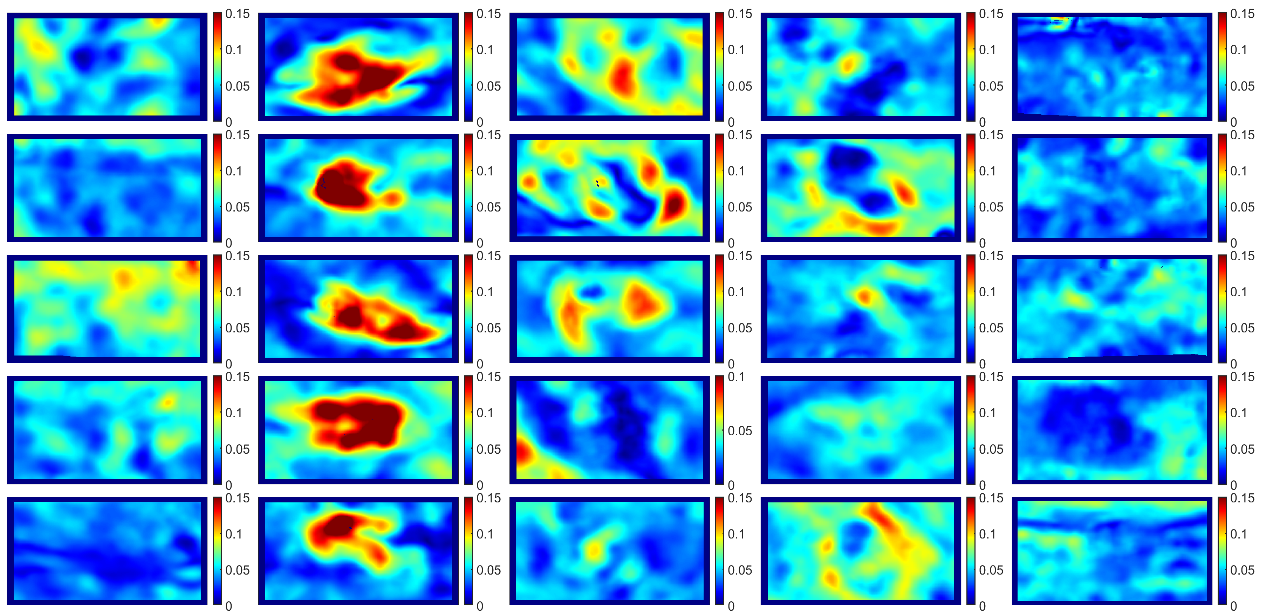

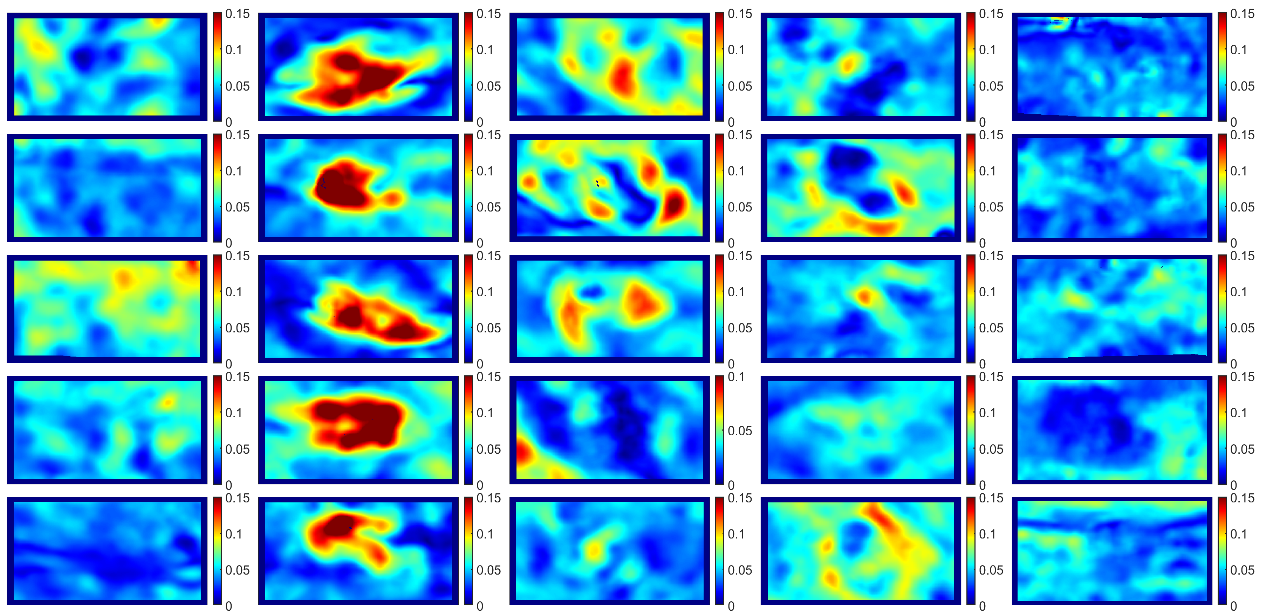

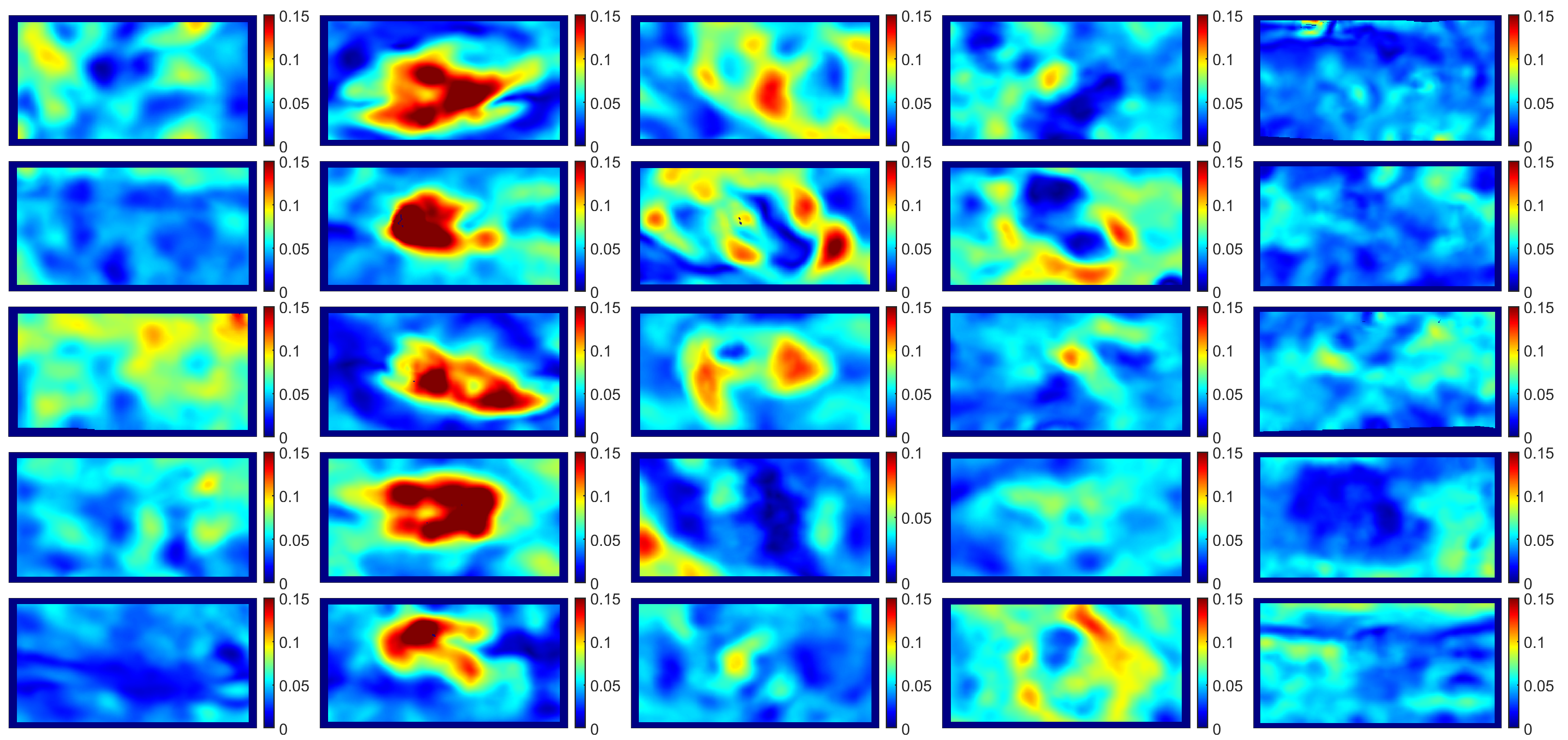

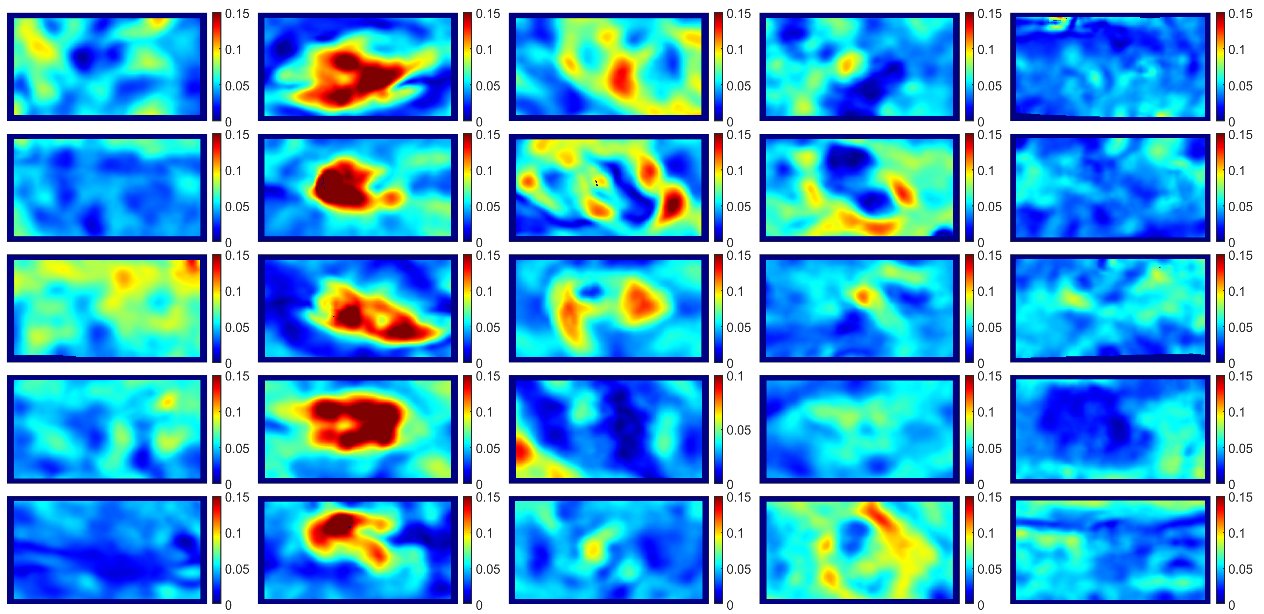

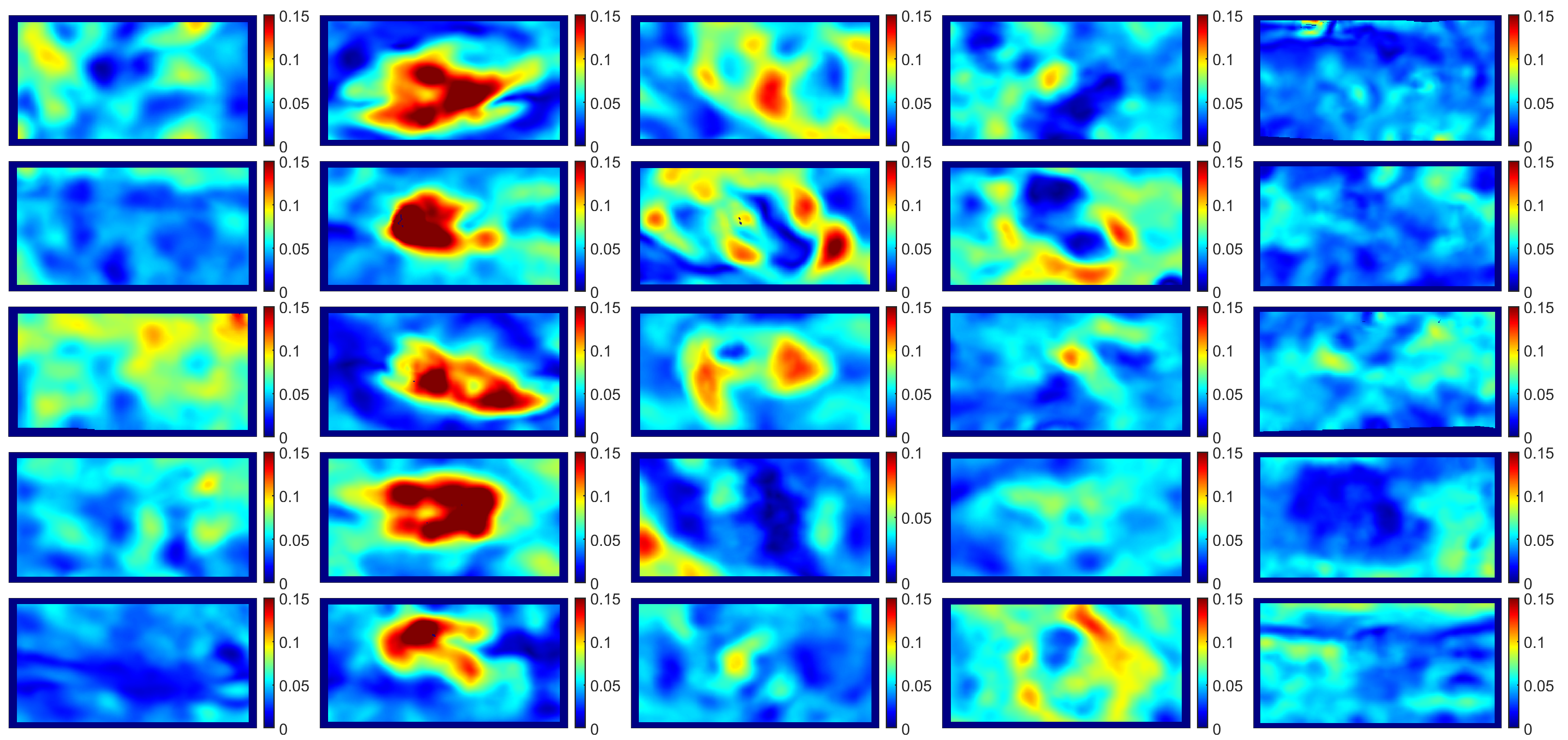

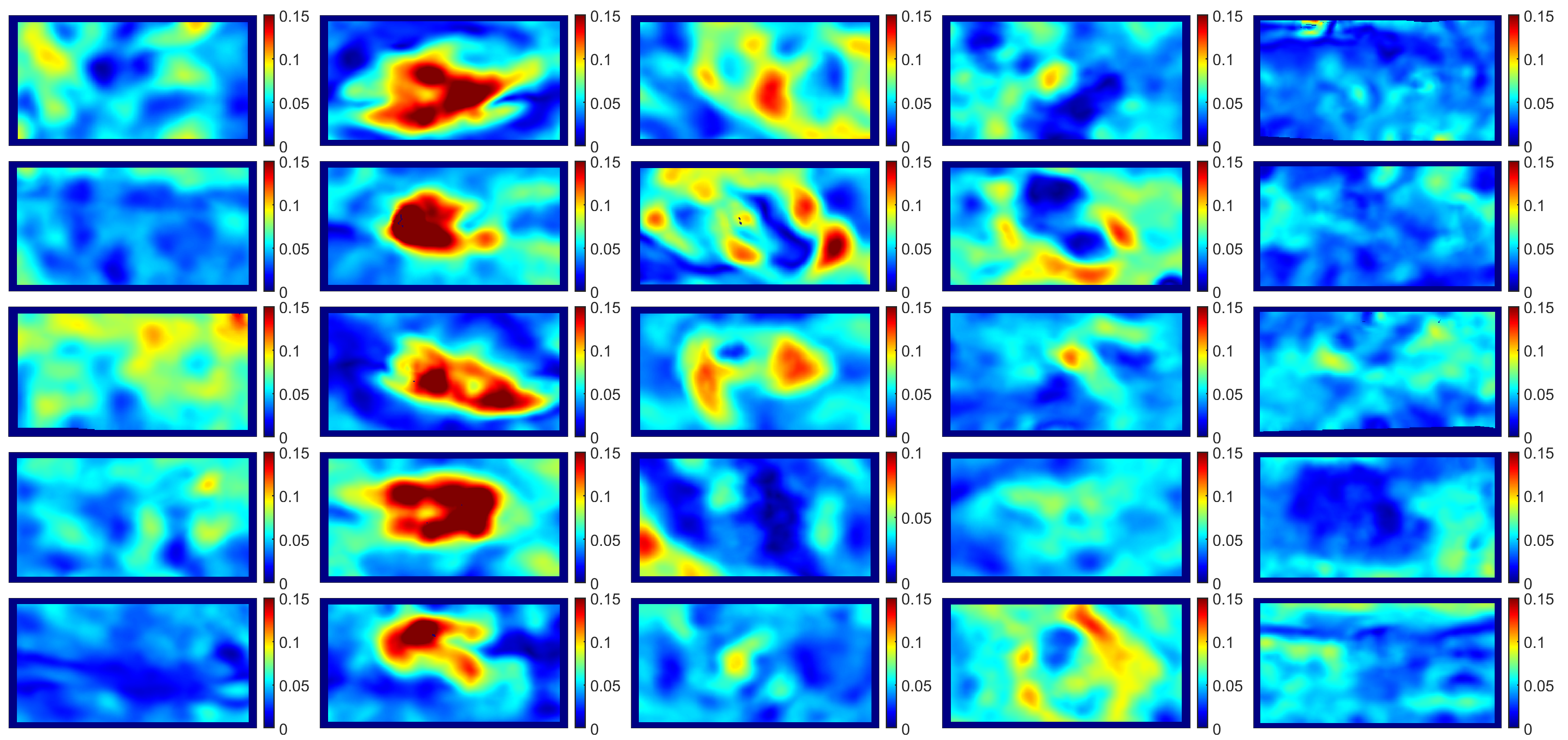

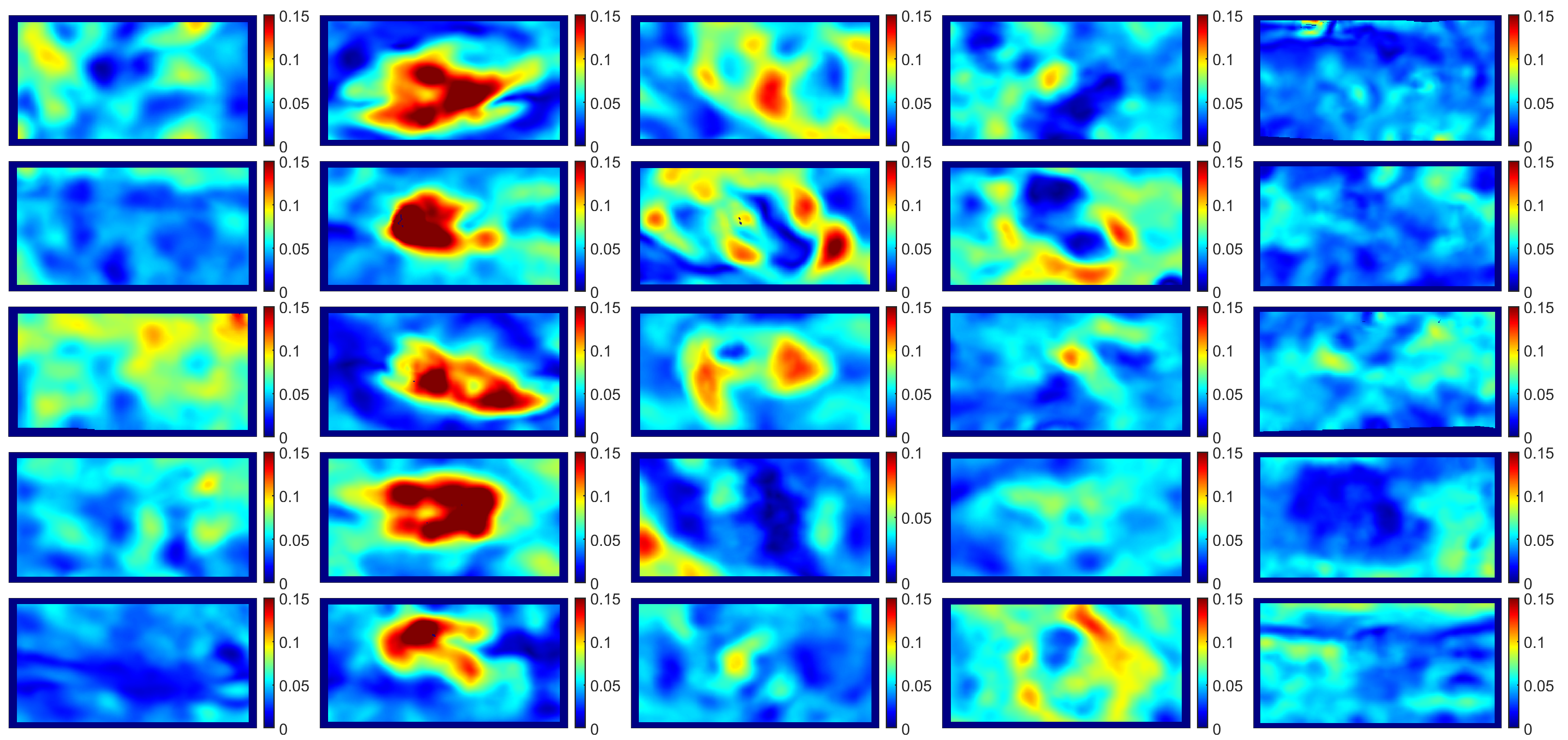

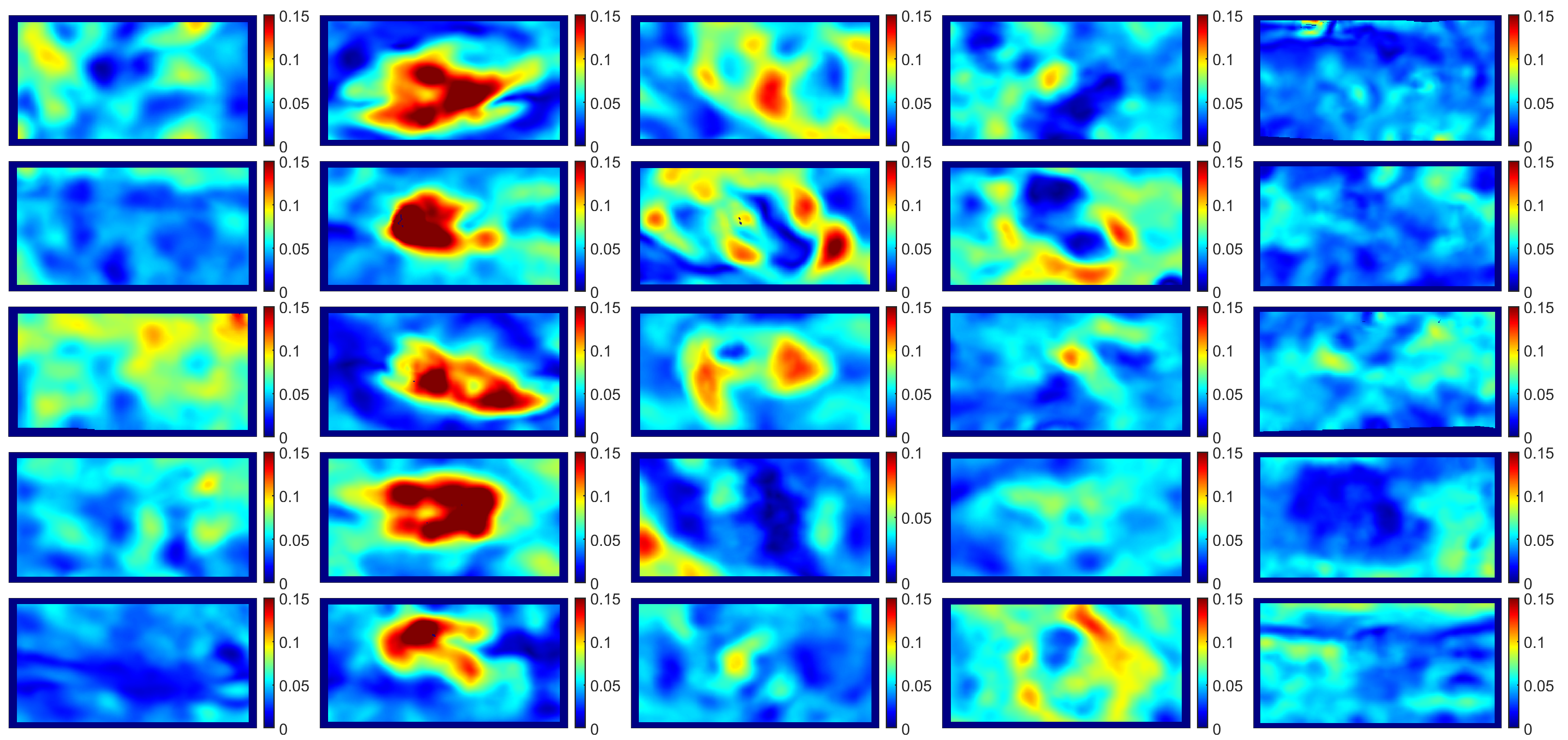

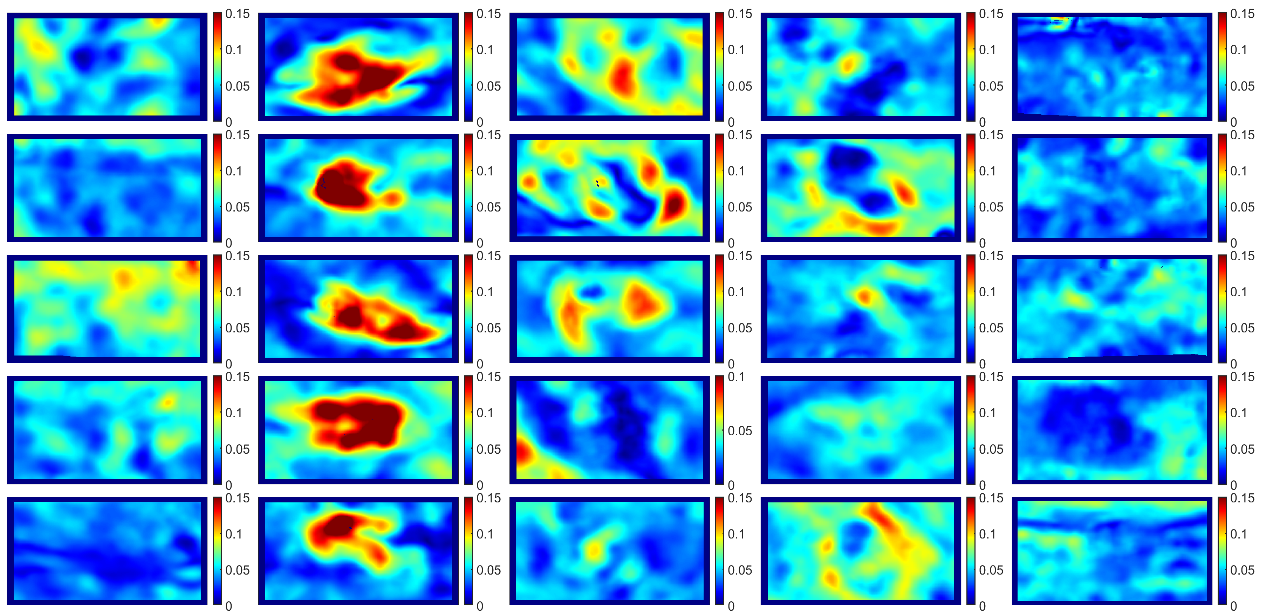

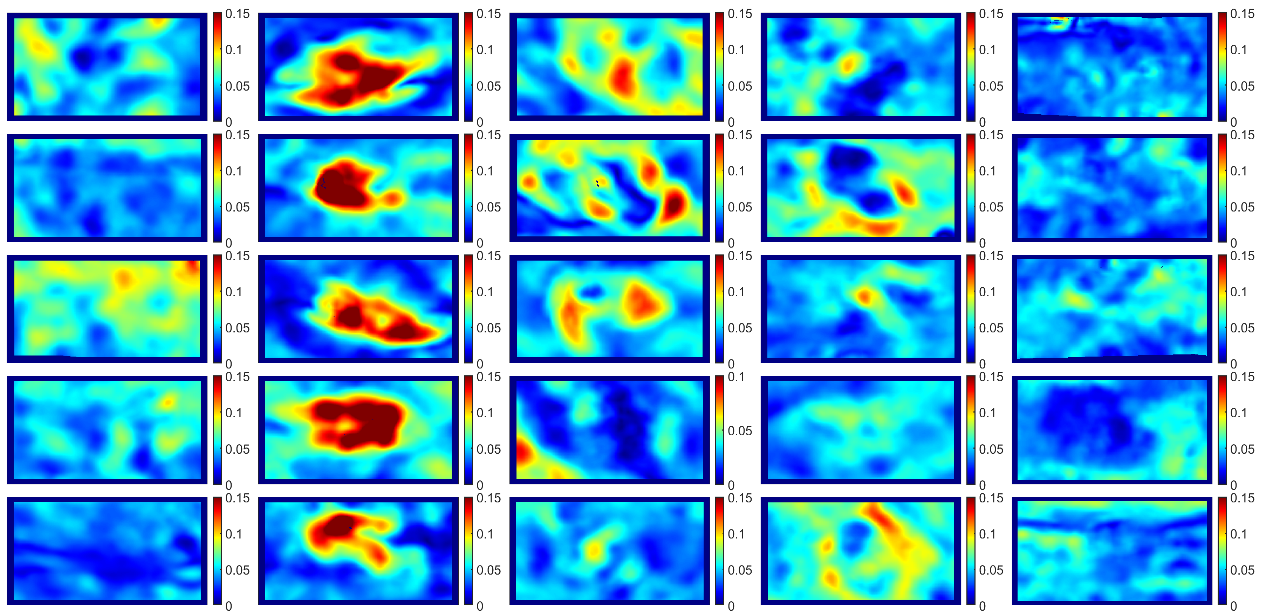

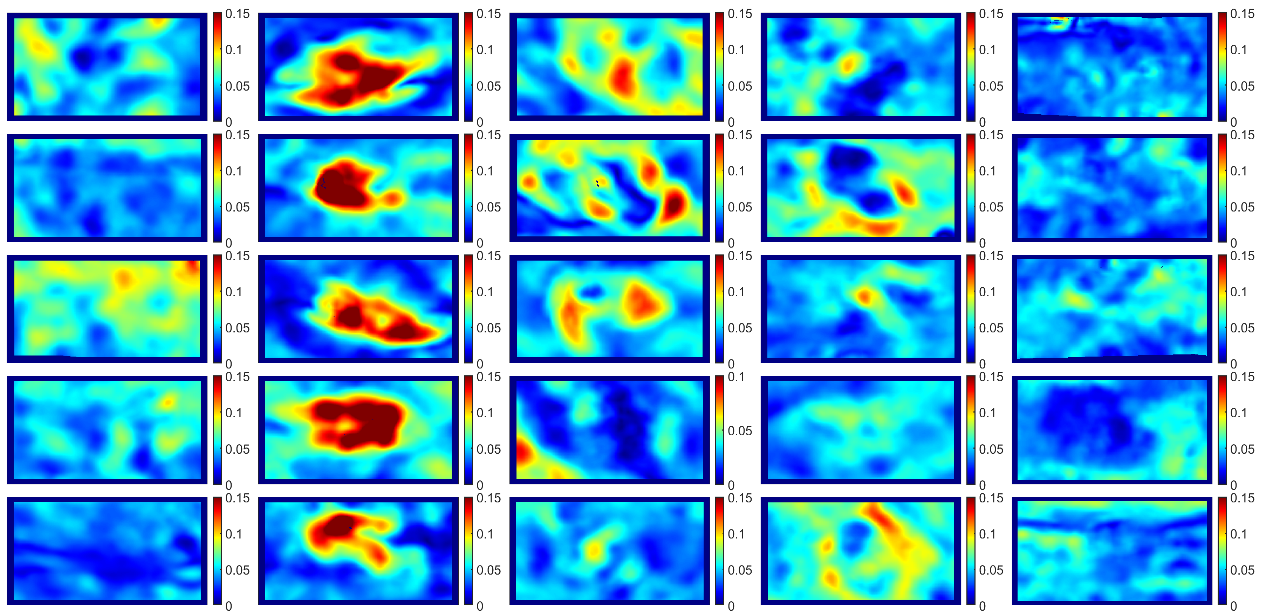

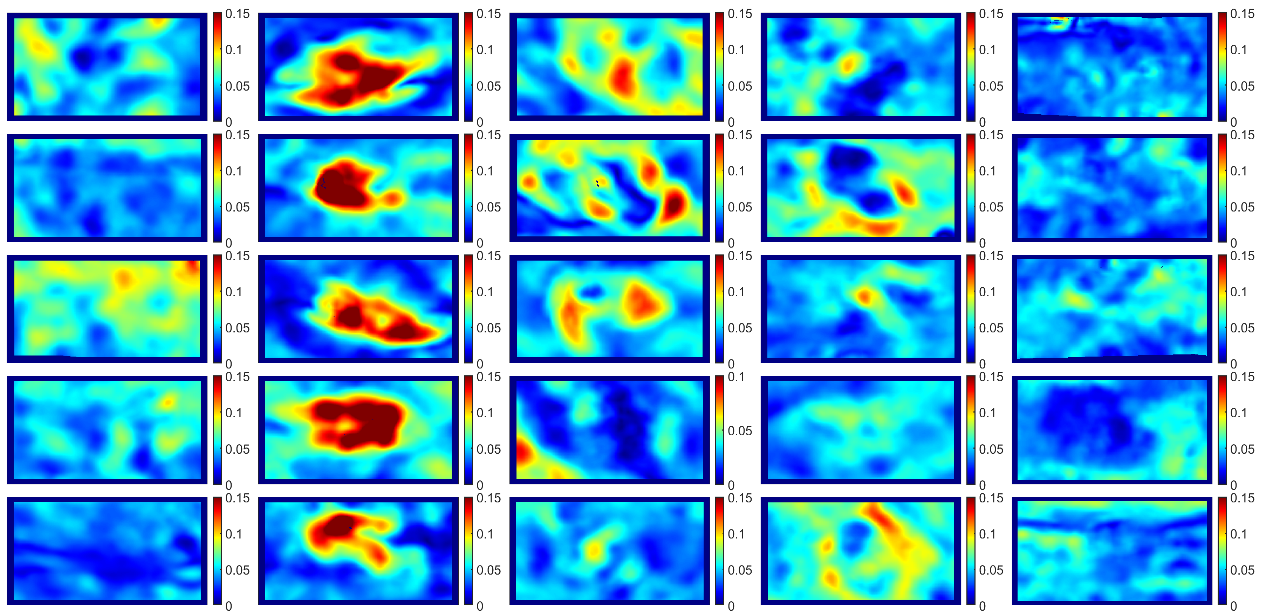

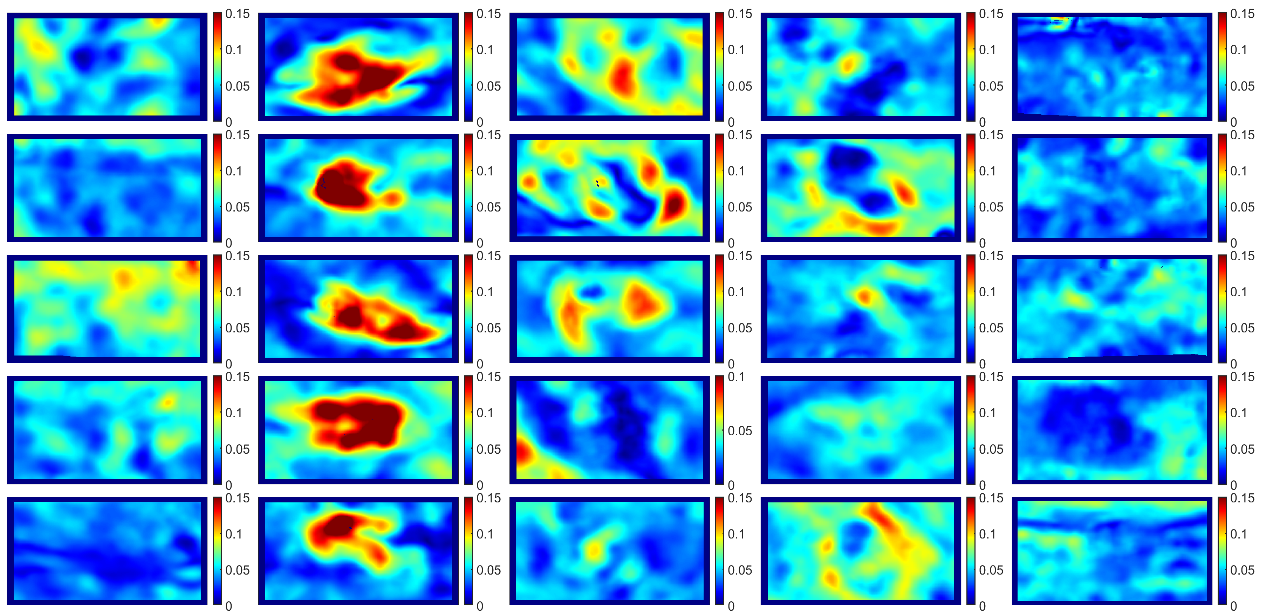


***Fig. S9. An attempt to overlay all strain maps from days 1, 3 and 7***

Despite this, in **Fig. 9** an evolution of the overall cohort is still evident, as the peaks of maximum deformation (which some authors have named or correlated with the “functional wound length”) narrow, until they disappear by day 14. Future studies will look at implementing better identification systems for these features (e.g., by using machine learning algorithms) to better classify each wound; however, we believe that the current analysis is a good starting point to determine that physiological changes have attached a mechanical signature that – with further refinements – can be non-invasively characterised and used in clinical settings.

**The codes used to extract the data from the strain maps for creating all figures, as well as the raw data (i.e., both DIC and tensile testing results) will be made available upon request (m.crichton@hw.ac.uk).*

1. **Alignment coefficient: local vs global analysis**

The local alignment coefficients vs global are summarised in **table S5** and plotted **in Fig**. S10.

**Table S5. Alignment coefficients extracted from the different regions of interest (wound adjacent and far field) and from the global analysis.** The averages and standard deviations are shown at the end of each group’s column.

|  | **Wound edge** | **Far from wound** | **Global Analysis** |
| --- | --- | --- | --- |
| **Day 1** | 0.63214 | 0.34371 | 0.40079 |
|  | 0.61447 | 0.37843 | 0.32394 |
|  | 0.58179 | 0.39186 | 0.51642 |
|  | **av. 0.61** | **av. 0.37** | **av. 0.41** |
|  | **std. 0.02** | **std. 0.02** | **std. 0.1** |
| **Day 3** | 0.40356 | 0.36423 | 0.34754 |
|  | 0.3175 | 0.22538 | 0.31667 |
|  | 0.50504 | 0.28305 | 0.27136 |
|  | **av. 0.41** | **av. 0.29** | **av. 0.31** |
|  | **std. 0.09** | **std. 0.07** | **std. 0.04** |
| **Day 7** | 0.37912 | 0.1711 | 0.20826 |
|  | 0.26366 | 0.23865 | 0.11459 |
|  | 0.19149 | 0.11653 | 0.25305 |
|  | **av. 0.28** | **av. 0.17** | **av. 0.19** |
|  | **std. 0.09** | **std. 0.06** | **std. 0.07** |
| **Day 14** | 0.16287 | 0.11876 | 0.05933 |
|  | 0.18795 | 0.09876 | 0.05468 |
|  | 0.15437 | 0.13196 | 0.03460 |
|  | **av. 0.17** | **av. 0.12** | **av. 0.05** |
|  | **std. 0.02** | **std. 0.02** | **std. 0.01** |


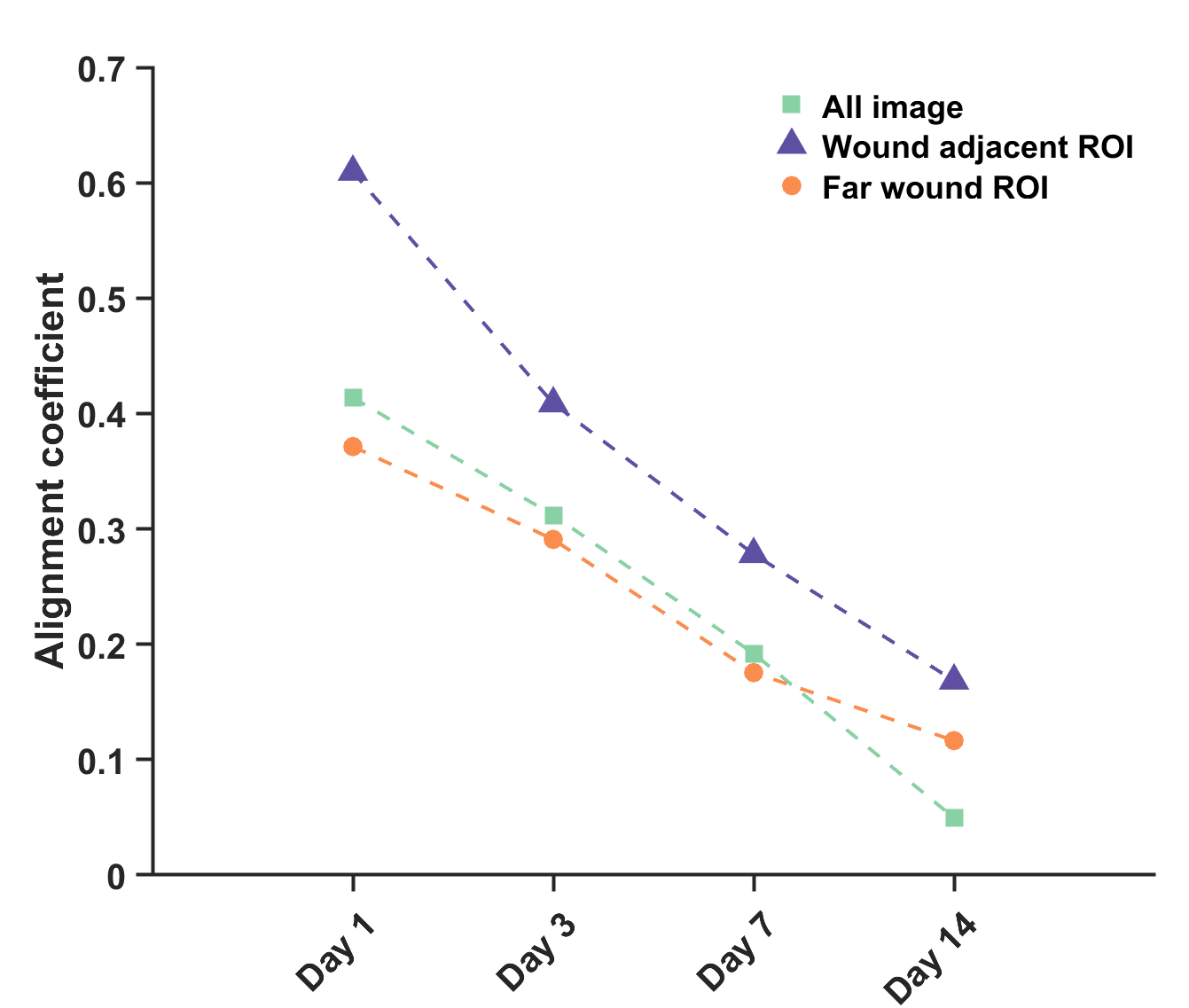


***Fig. S10. Global vs local alignment coefficients***

**REFERENCES**

[1] W. Craig, ‘Novel Patterning Techniques to Improve Digital Image Correlation in Challenging Environments’.

[2] K. Mallett, ‘Experimental and Computational Characterization of the Anterior Cruciate Ligament: Challenges and Considerations for Soft Tissue Biomechanics’, Thesis, 2017. Accessed: Jan. 19, 2023. [Online]. Available: http://deepblue.lib.umich.edu/handle/2027.42/138606

[3] ‘Picrosirius Red Staining Protocol for Collagen’. Accessed: Nov. 17, 2022. [Online]. Available: http://www.ihcworld.com/_protocols/special_stains/sirius_red.htm

[4] L. Rittié, ‘Method for Picrosirius Red-Polarization Detection of Collagen Fibers in Tissue Sections’, in *Fibrosis: Methods and Protocols*, L. Rittié, Ed., in Methods in Molecular Biology. , New York, NY: Springer, 2017, pp. 395–407. doi: 10.1007/978-1-4939-7113-8_26.

[5] R. Lattouf *et al.*, ‘Picrosirius Red Staining: A Useful Tool to Appraise Collagen Networks in Normal and Pathological Tissues’, *J. Histochem. Cytochem.*, vol. 62, no. 10, pp. 751–758, Oct. 2014, doi: 10.1369/0022155414545787.

[6] F. Faul, E. Erdfelder, A.-G. Lang, and A. Buchner, ‘G*Power 3: A flexible statistical power analysis program for the social, behavioral, and biomedical sciences’, *Behav. Res. Methods*, vol. 39, no. 2, pp. 175–191, May 2007, doi: 10.3758/BF03193146.
